# Monitoring the Antioxidant Mediated Chemosensitization and ARE-Signaling in Triple Negative Breast Cancer Therapy

**DOI:** 10.1371/journal.pone.0141913

**Published:** 2015-11-04

**Authors:** Kira Foygel, Thillai V. Sekar, Ramasamy Paulmurugan

**Affiliations:** Molecular Imaging Program at Stanford, Bio-X Program, Stanford University School of Medicine, Stanford, California, United States of America; Wayne State University School of Medicine, UNITED STATES

## Abstract

Chemotherapy often fails due to cellular detoxifying mechanisms, including phase-II enzymes. Activation of Nrf2-Keap1 pathway induces phase-II enzymes expression through ARE-signaling and prevents cancer development. Nrf2-overexpression in cancer cells results in chemo- and/or radioresistance. This necessitates understanding of Nrf2-regulation, and identification of Nrf2 activators/inhibitors sensitizing cancer cells to improve chemotherapy. N-terminal 435-amino acids of Nrf2 are crucial for Keap1 binding during ubiquitination. Identification of a minimum Nrf2-domain required for Keap1 binding without altering endogenous ARE-signaling would be a novel tool to study Nrf2-signaling. Current study developed firefly-luciferase reporter fusion with N-terminal Nrf2-domain of different lengths and examined its response to Nrf2-activators in cells. The results identified FLuc2 fusion with N-terminal 100-aa of Nrf2 is sufficient for measuring Nrf2-activation in cancer cells. We used MDA-MB231 cells expressing this particular construct for studying antioxidant induced Nrf2-activation and chemosensitization in triple-negative breast cancer therapy. While antioxidant EGCG showed chemosensitization of MDA-MB231 cells to cisplatin by activating Nrf2-ARE signaling, PTS, another antioxidant showed chemoprotection. Tumor xenograft study in mouse demonstrates that combinational treatment by cisplatin/EGCG resulted in tumor growth reduction, compared to cisplatin alone treatment. The results of this study highlight the importance of identifying selective combination of antioxidants/chemotherapeutic agents for customized treatment strategy.

## Introduction

Chemotherapy is ubiquitous for treating different cancers, including triple negative breast cancer, which has no targeted therapy. Unfortunately, chemotherapy often fails due to the resistant nature of tumors. Three major mechanisms protect cells from inadvertent exposure to toxic chemicals, which include, 1) multidrug resistant efflux pump of cell membranes, 2) phase I neutralizing enzymes (cytochrome p450), and 3) phase II detoxifying enzymes. In cancer therapy, all three protective mechanisms hinder therapy response from cancer cells. However, though most of the currently used anticancer drugs are designed to overcome the first two mechanisms, the phase II detoxifying enzymes are more potent and block therapeutic actions of anticancer drugs. NF-E2-related factor-2 (Nrf2) is a transcription factor that regulates phase II enzymes expression and controls the action of anticancer drugs in cancer therapy. Nrf2 is ubiquitously expressed at low levels in all human tissues. A stringent regulation of Nrf2 is crucial for maintaining cellular homeostasis and preventing many human diseases, such as cancer, neurodegenerative disorders, cardiovascular diseases, ischemia, diabetes, pulmonary disease, and inflammatory diseases [1, 2].

**Fig 1:**
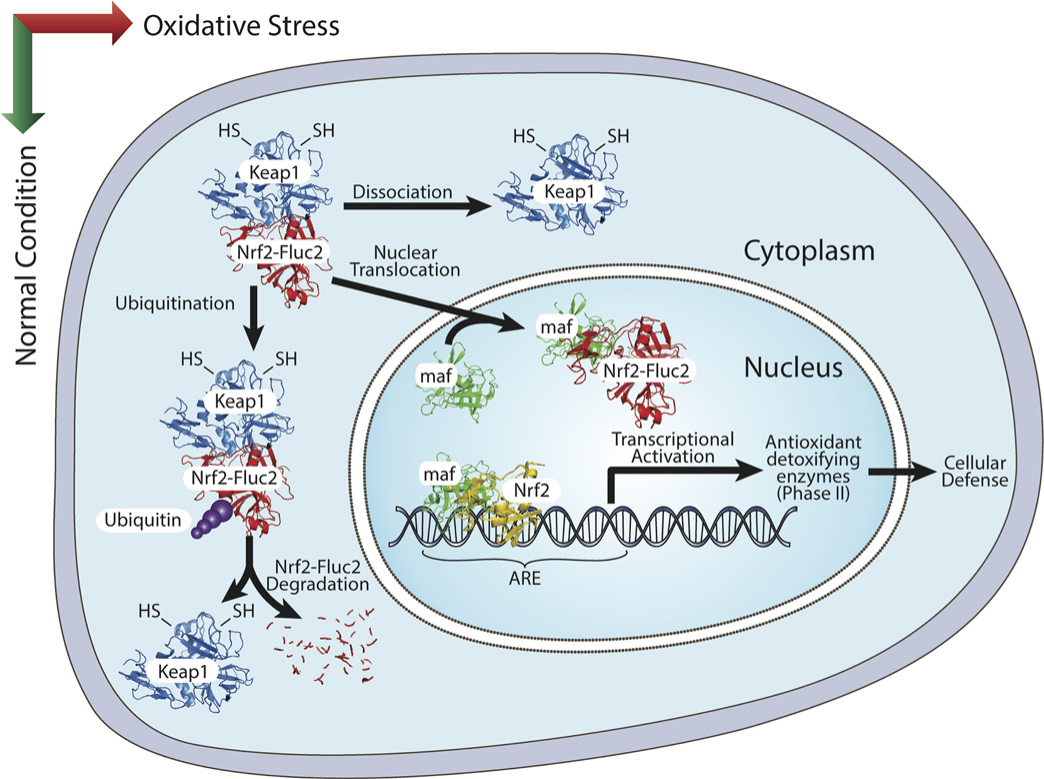
Schematic illustration of Nrf2 pathway in cells and Nrf2-FLuc2 construct, the measure of Nrf2 nuclear translocation in cells in response to its activators.

Nrf2 protein is maintained in the cytoplasm as an inactive complex by binding to a repressor molecule known as Keap1 (Kelch-like ECH-associated protein-1). This process facilitates Nrf2 ubiquitination and maintains its cellular level constant. During redox stress, cytosolic Nrf2 is phosphorylated by protein kinase C and Map kinase [3], which translocates Nrf2 in to nucleus where it activates downstream target genes expression through antioxidant response elements (AREs). Reactive oxygen species (ROS), free radicals, and electrophiles produced by cells in response to environmental exposure to chemical toxicants play significant roles in developing major cellular disorders. Nrf2 acts as a gatekeeper by protecting cells from these stress-induced disorders by activating several downstream genes, such as glutathione S-transferase (GST), quinone reductase, epoxide hydrolase, heme oxygenase (HO), UDP-glucuronosyl transferases, and gamma-glutamylcysteine synthetase. It has been demonstrated that expression of Nrf2-target genes protects cells from oxidative damage, and prevent mutagenesis and cancer development (**Fig 1**). On the other hand, constitutive activation of Nrf2 and high expression of its downstream target genes has been reported in many primary tumors and in cancer cell lines [4]. Overexpression of Nrf2 in cancer cells protects them from the cytotoxic effects of anticancer therapies, resulting in chemo- and/or radioresistance [5].

Overproduction of reactive oxygen species in cells may amplify the pro-inflammatory state of diseased areas culminating in oxidative stress and loss of cellular functions [6]. The use of antioxidants to boost the Nrf2-dependent adaptive response to counteract environmental insults has emerged to be a promising strategy for cancer prevention and in treating metabolic disorders such as type-1 and type-2 diabetes [7, 8]. However, recent emerging data has revealed the “dark” side of Nrf2 in cells [5, 9]. The discovery of the dual role of Nrf2 (redox signaling and apoptotic regulation) in cells has brought some safety concerns with respect to the use of Nrf2 activators for disease prevention; however, in normal cells, Nrf2 is under tight regulation by the Keap1-Cul3E3 ligase [10, 11]. In response to activators, Nrf2 is transiently induced, and functional Keap1 will reduce Nrf2 to basal levels once redox balance is restored.

The Nrf2 protein in humans is 605 amino acids long and contains seven highly conserved regions known as Nrf2-ECH homology (Neh) domains. Neh1 contains the CNC–bZIP domain, which mediates heterodimerization with Maf. The Neh2 domain contains the two degrons that are specifically bound by Keap1. These are commonly known as the DLG and ETGE motifs after their sequence conservation in the single-letter amino acid code. Two redox-independent degrons have been described in Neh6. These motifs are not recognized by Keap1, but are instead targeted for degradation by the E3 ubiquitin ligase β-TrCP. This alternative pathway is enhanced by the phosphorylation of Nrf2 by glycogen synthase kinase-3β, providing another mechanism for cellular control of Nrf2 activities. The Neh3–5 domains are thought to function in transactivation by binding to various components of the transcriptional apparatus. Conversely, repression of Nrf2 is conferred by the interactions of Neh7 with the DNA-binding domain of retinoic X receptor α [12]. Two nuclear localization signal (NLS) motifs in Nrf2 have been identified, one located near the N-terminal region (amino acid residues 42–53) and the other (residues 587–593) located near the C-terminal region [13].

The activation of Nrf2 is a potential strategy to overcome a variety of diseased states, but there are not many accurate sensing systems available to detect Nrf2 activation in cells or non-invasive imaging in living animals. The N-terminal 435 amino acids of Nrf2 are crucial for Keap1 binding, though, the exact length of N-terminal binding site required for Keap1 interaction is not known. We therefore asked the question of how shortening the Nrf2 sequence would reflect upon Nrf2-ARE signaling in response to activators without showing much impact on the endogenous Nrf2 target genes activation. In the current study, we constructed vectors expressing fusion proteins expressing N-terminal Nrf2 of different lengths (50, 100, 150, 200, 250, 350 and 435 amino acids long) fused to luciferase (Nrf2-FLuc2). We attempted to establish the N-terminal amino acid fragment length that would be short enough as to not alter the endogenous Nrf2 functional mechanism and at the same time, retain the binding ability with Keap1. Since understanding Nrf2 regulation, and identifying Nrf2 activators or inhibitors that may play a role in disease prevention or cancer cell sensitization during therapy, would have a significant impact on cancer therapy, we studied these constructs in MDA-MB231 triple negative breast cancer cells stably expressing Nrf2-FLuc2 fusion in response to an array of Nrf2 activators and measured for the luciferase activity. Upon treatment Nrf2-FLuc2/Keap1 complex dissociates, and the fusion protein (Nrf2-FLuc2) translocates into the nucleus and escapes from ubiquitination. The measured increase in luciferase enzyme level is dependent on the effectiveness of Nrf2 translocation into the nucleus. The results showed N-terminal Nrf2 of as short as 100 aa length is sufficient to maintain the structural information necessary for successful Keap1 binding and ubiquitination. We used the developed Nrf2-FLuc2 fusion protein to measure Nrf2-activator mediated redox signaling during chemotherapy in cancer cells. We paid particular attention to monitoring the switches from protective redox signaling in to an apoptotic signaling which occurs as a result of co-treatment with Nrf2 activators and chemotherapeutic drugs, by simultaneously measuring the redox effect and therapeutic efficiency. The results of this study identified that Nrf2-activators can synergistically enhance therapeutic effect of chemotherapeutic drugs if a correct combination of anticancer drug with Nrf2 activator is identified. Importantly, despite the justified fears that such synergistic combinations may be cytotoxic to other cells [14], we have shown that the identification of correct combination of Nrf2 activator with chemotherapeutic drug produces little to no toxicity in normal cells.

## Materials and Methods

All experiments were carried out in accordance with the approved guidelines.

### Construction of plasmids

The Nrf2-FLuc2 fusion sensors were constructed by PCR cloning. The N-terminal Nrf2 of different lengths were PCR-amplified using the forward primer flanking 1–21 N-terminal nucleotides with Nhe I restriction enzyme site and reverse primers flanking the specific positions of each truncated regions of N-terminal Nrf2 domain with Bam HI restriction enzyme. The Nhe I/Bam HI restriction enzyme-digested fragments of the different lengths (150, 300, 450, 600, 750 and 1,050 bp) of Nrf2 were cloned into a modified version of pcDNA3.1(+) vector backbone (Invitrogen, Carlsbad, CA) [Neomycin was replaced with Puromycin] which express FLuc2 enzyme cloned in BamH1/XhoI restriction enzyme site. The sequence-confirmed vectors of different constructs were used for various experiments.

### Experimental cell lines

All cell lines used in this study were purchased at ATCC (Manassas, VA), and were mycoplasma free as determined by regular testing.

### Cell transfection and Luciferase assay

To find the optimal Nrf2 length, the constructed eukaryotic plasmid vectors expressing fusion proteins of full length firefly luciferase (FLuc2) with different Nrf2 fragments were transiently transfected into MDA-MB231 human triple negative breast cancer cell line, and after 24h firefly luciferase activities were measured after exposure to Nrf2 activators for various time points. The cells were co-transfected with vector express Renilla-luciferase under a constitutive ubiquitin promoter to normalize transfection efficiency. The luminometry assays for FLuc and RLuc activity were performed as previously described [15]. In brief, transfected cells were lysed in 200 μl of ice-cold 1X passive lysis buffer (Promega, Madison, WI) by shaking for 10 min, and cell lysates were centrifuged for 5 min at 10,000 g at 4°C to remove cell debris. To determine the FLuc activity, 10 μl of supernatant was assayed by photon count for 10 seconds in luminometer (model T20/20, Turner Designs, Sunnyvale, CA), after the addition of 50 μl of LARII substrate (Promega, Madison, WI). To determine the co-transfected RLuc activity, 10 μl of supernatant was assayed by photon count for 10 seconds in luminometer after the addition of 100 μl of 10μg/ml coelenterazine substrate (Nanolight, Pinetop, AZ). Protein concentrations from cell lysates were determined by using the Bradford protein assay reagent (Bio-Rad Laboratories Inc., Hercules, CA). Luciferase activities were expressed as fold induction relative to values obtained from control cells after normalization to Renilla-luciferase activity and protein concentration. The results represent mean of at least three independent transfection experiments.

### Constructing stable MDA-MB231 cells expressing Nrf2-FLuc2 fusion constructs

Transfections were performed in 80% confluent 24 hours old cultures of MDA-MB231 cells. For transfection, 10 μg of Nrf2-FLuc2 plasmid DNA and Lipofectamine-2000 in 1:3 ratio were used by following the manufacturer’s instructions (Invitrogen, Carlsbad, CA). After 24 hours, media was supplemented with 1 μg/ml of puromycin, and surviving cell population was further expanded; these steps were repeated until no further cell death was observed. The stable cells were used for all further experiments.

### Evaluation of Nrf2-FLuc2 activation in response to Nrf2 activators

To evaluate the efficacy of the Nrf2-FLuc2 activation, several known Nrf2 activators [resveratrol (RES), pterostilbene (PTS), thapsigargin (THA), epigallocatechin gallate (EGCG), sodium arsenite (NaAs), di-ethyl maleate (DEM), methyl methane sulphonate (MEM) were used to treat MDA-MB231 cells stably expressing Nrf2-FLuc2 constructs. The compounds at different concentrations were tested under optimized conditions for 8–12 hours. The cells were harvested and assayed for luciferase activity by luminometry.

### Immunoprecipitation and Immunoblotting

Nrf2-FLuc2 and FLuc2 constructs transiently transfected in HeLa cells, or MDA-MB231 cells were grown in 6-well tissue-culture plates t0 70% confluency, and were treated with Nrf2 activators and/or chemotherapeutic dugs as required by experimental design. To obtain total lysate, cells were resuspended in lysis buffer [50 mM Tris, pH 7.5, 150 mM NaCl, 1% Nonidet P-40, 5 mM EDTA, protease inhibitors (Roche, Pleasanton, CA) and lysed by keeping on ice for 30 min with intermittent mixing. Soluble cell lysates of 30–50 μg of total protein were resolved in 4–12% gradient polyacrylamide gel by electrophoresis, and electroblotted onto a nitrocellulose membrane (Schleicher & Schuell, Keene, NH) of 0.2-μm pore size. The membranes were then exposed to the primary antibodies, as required by experimental design, i.e. anti-Nrf2, NQO1 (1:200 dilution, Santa Cruz Biotechnologies, Santa Cruz, CA), anti-Fluc, PARP and p53 (1:200 dilution, Cell Signaling, Boston, MA) or anti-GST1 (1:200 dilution, GenScript, Piscataway, NJ), followed by HRP conjugated secondary antibodies (1:5,000 dilution, Jackson ImmunoResearch, West Grove, PA), and the membranes were then visualized using ECL system (Thermo Scientific, San Jose, CA) in a optical CCD camera imaging (IVIS). Anti-GAPDH antibody (1:500 dilution, Cell Signaling, Boston, MA) was used for loading control determinations, and the signals were quantified using Live Image software (Perkin-Elmer, Pittsburg, PA). For immunoprecipitation, 50 μg of total protein extracted from MDA-MB231 cells transiently transfected with either Nrf2-100-Fluc2 plasmid or Fluc2 plasmid and treated with and without 5 μM PTS overnight lysed in Co-IP lysis buffer (ThermoFisher), was diluted with PBS to 1 ml volume. The sample was mixed with anti-luciferase (mouse monoclonal antibody, Santa Cruz Biotechnologies, Santa Cruz, CA) at 1:200 dilution and incubated at 4°C overnight, with gentle mixing. To pull down the Keap1-Nrf2-100-Fluc2 complex, 10 μl of Dynabeads-coupled streptavidin (Life Technologies AS, Norway) was added to each sample and incubated further for 2 h with gentle mixing. The Dynabeads coupled with Keap1-Nrf2-100-Fluc2 complex were washed three times with PBS using a magnetic stand, and resuspended in PBS. The resuspended protein was resolved by 4–12% gradient SDS/PAGE (Invitrogen) and electroblotted onto a 0.2 μM pore size nitrocellulose membrane (Schleicher & Schuell). Pre-stained protein marker was used to confirm the molecular mass and complete transfer of protein to the membrane. The membrane was blocked in 5% non-fat dry milk in TBS-T (TBS with 0.05% Tween 20) buffer for 1 h and further incubated in the same blocking solution with rabbit anti-Keap1 antibody (Santa Cruz Biotechnologies, Santa Cruz, CA), 1:200 diluted, overnight at 4°C on a rotating platform. The membrane was washed 3 times with TBS-T and incubated with goat anti-rabbit HRP-conjugated antibody (1:5,00 dilution) for one hour at room temperature. The membrane was washed 3 times with TBS-T buffer before incubation with the chemiluminescent HRP substrate LuminoGlo (Cell Signaling, Beverly, MA), following the manufacturer's instructions. The signal was detected using IVIS optical CCD camera imaging and drawing ROI over the bands signal was quantified.

### Immunofluorescence staining for Luciferase

For immunofluorescence staining, cells were grown on glass cover slips and transiently transfected with Nrf2-100-Fluc2 or Fluc2 plasmid, and exposed to 5 μM PTS overnight. Unexposed transfected cells served as controls. The cells were fixed in 4% paraformaldehyde prepared in phosphate-buffered saline (PBS) for 10 minutes and permeabilized with 0.05% Triton X-100 in PBS for three hours. Cells were then blocked in PBS containing 1% bovine serum albumin and incubated with primary anti-luciferase mouse monoclonal antibody (Santa Cruz Biotechnologies, Santa Cruz, CA) overnight at 4°C (1:100 dilution), washed and incubated with goat anti-mouse Alexa Fluor 488 (Invitrogen, Carlsbad, CA) (1:2000 dilution) for 1 hour. The nuclei were counterstained with DAPI, the glass cover slips were washed in PBS and mounted in anti-fade media. Confocal images were obtained by using a Leica SP2 AOBS laser scanning confocal microscope.

### ARE-Luciferase Reporter Gene Assay

hNQO1-ARE-Luc and GST-ARE-Luc reporter gene constructs used for cell-based reporter gene assay were provided by Donna D. Zhang (College of Pharmacy, University of Arizona, Tucson, AZ)^26^. ARE-Luc construct (500 ng/well) was transiently transfected into HeLa cells in 24-well plates using Lipofectamine 2000 as per manufacturer’s instruction. 48 hours after transfection, media was changed, Nrf2 activators were added at concentrations previously determined, and cells were incubated for another 12 hours. The luminometry assay was performed as previously described. Luciferase activities were expressed as fold induction relative to values obtained from control cells. The results represent the mean of at least three independent transfection experiments.

### Treatment conditions and apoptosis by PI-staining based FACS analysis

For FACS analysis, cells were plated in 12-well plates at 60–70% confluency in DMEM medium supplemented with 10% FBS. Cells were grown overnight and changed to DMEM medium supplemented with 10% FBS and exposed to the required conditions (e.g. various drug, antioxidant or drug/antioxidant combination at different concentrations), each condition being represented by three wells; unexposed cells served as control in every experiment. Cells were then allowed to grow for 24, 48, 72 and 96 hours post exposure. All cells were collected by trypsinization and resuspended in 0.5 mL of ice cold PBS, and then fixed by adding 2 ml of 100% ice-cold ethanol. The samples were stored at -20°C until FACS analysis. All samples from a given experiment (24–96 hours) were analyzed at the same time. Briefly, cells were spun, washed once in PBS, and resuspended in 0.5 ml of PBS/RNaseA (10 μg/ml)/Triton X-100 (0.1%) buffer containing 0.5 μg/ml of propidium iodide (PI). After 15 min incubation period at room temperature, cells were subjected to FACS analysis (BD FACSAria^TM^ III), and generated data were analyzed by FlowJo 8.8.6 software.

### MDA-MB231 cells siRNA transfection for FACS and immunoblot analysis

MDA-MB231 cells were grown to 70% confluency in 6-well plates, transfected with 30 μM of either scrambled or Nrf2 siRNA (Santa Cruz Biotechnologies, Santa Cruz, CA) using Lipofectamine-2000 ((Invitrogen, Carlsbad, CA), for 24 hours, and were then exposed to either cisplatin, EGCG or the combination for 48 hours (untreated cells served as control). Cells were then collected for either FACS or immunoblot analysis, as described above.

### Animal Experiments

All animal experiments were carried out under the guidance of the Administrative Panel on Laboratory Animal Care (APLAC), Stanford University. The Institute animal research committees at Stanford approved all animals handling. All animals (CD1, nude, females) were purchased from Charles River laboratories (Wilmington, MA).

#### In vivo tumor growth evaluations

The MDA-MB231 cells were used for the studies of combinational treatment (chemotherapeutic drug/antioxidant) therapeutic effect. Twenty mice were implanted in the left and right flanks (3x10^6^ cells) (n = 40 tumors) using 50% (vol/vol) Matrigel (100 μL total volume injected). Animals were anesthetized by 2% isoflurane in oxygen gas during the implantation procedure. Animal health and tumor size were monitored daily. Tumors were allowed to grow to about 100–200 mm^3^ size, and then mice were randomly assigned to one of four groups of 5 animals each (n = 10 tumors) according to treatment conditions: (a) control (vehicle-treated), (b) cisplatin-treated (5 mg/kg), (c) EGCG-treated (100 mg/kg), (d) cisplatin-EGCG treated. The animals were treated according to the conditions described above on Days 0, 5, and 11, and the tumors were measured on Days 1, 5, 6, 11,12 and 14.

#### Ex-vivo analysis

On Day 14 after treatment initiation, mice were euthanized by CO2 gas, and the tumors were excised and frozen in OCT cryoprotective fixing medium. Tumor xenografts were sliced at 10 μm in Leica cryomicrotome. Sections were stained in undiluted hematoxylene (Sigma-Aldrich, USA) for two min, rinsed in running water, and differentiated in 1% HCl acid/alcohol for 30 sec. They were then washed and immersed Bluing solution (Fisher, USA) for 1 min, washed in running water and rinsed in 10 dips of 95% alcohol. After this, slides were counterstained in eosin by dipping into 1:5 ethanol-diluted Eosin solution (Fisher) for a total of less than 30 sec, dehydrated through 95% alcohol, absolute alcohol, and xylene for 5 min each. Slides were mounted with xylene based mounting medium (Permount, Sigma) and imaged by Nanozoomer (Hamamatsu, Japan).

#### TUNEL assay

The tumor xenografts of MDA-MB231 cells were used to assess the combinational treatment therapeutic effects by measuring the apoptosis levels. Terminal deoxynucleotidyltransferase (TdT) nick-end labeling (TUNEL) assay was performed with a Trevigen TACS 2 TdT-DAB *in situ* Apoptosis Detection Kit (TREVIGEN, Gaithersburg, MD). A portion of the tumor was fixed with OCT (TissueTek), sectioned at 10 μm in a Cryomicrotome (Leica CM1850, Wetzlar, Germany). The tumor slices were processed for measuring apoptosis by following the manufacturer protocol (TREVIGEN, Gaithersburg, MD). After staining, the slides were scanned in a Nanozoomer 2.0 RS (Hamamatsu, Japan) digital scanner and viewed for Diaminobenzidine (DAB) staining of apoptotic tissues and cells using Nanozoomer digital pathology software.

### Statistical analysis

Data are presented as the means ± SEM. Differences were analyzed by unpaired two-tailed *t-*test between two groups. Statistical analysis was performed by one-way ANOVA, and *p*<0.05 was used to indicate a significant difference between groups.

## Results

### Construction of plasmid vectors expressing Nrf2-FLuc2 fusion with N-terminal Nrf2 of different lengths and their evaluation in response to the treatment of Nrf2 activators

**Fig 2:**
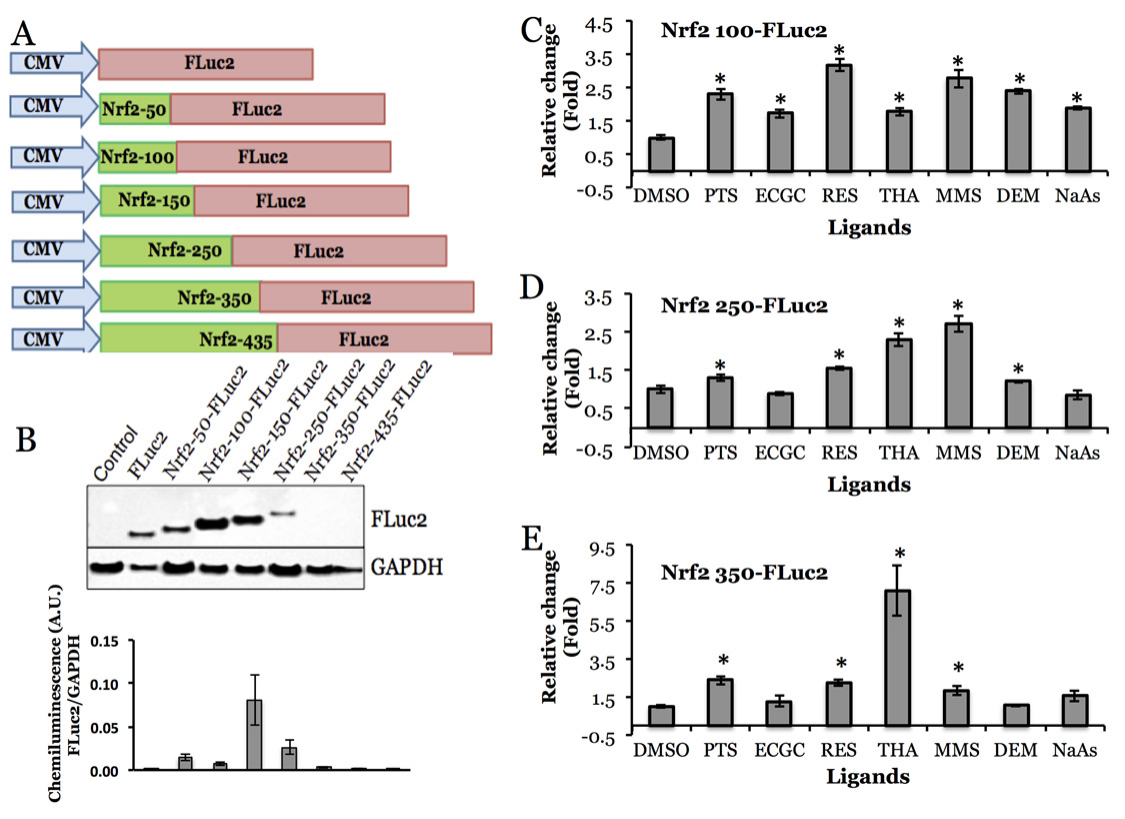
Construction and evaluation of Nrf2-Luciferase (Nrf2-FLuc2) truncation constructs in cells. A, Schematic illustration of Nrf2-FLuc2 truncation constructs. B, Expression of Nrf2-FLuc2 fusions in HeLa cells, immunoblot and its signal quantification normalized to GAPDH expression signal C, D, E, Activation of Nrf2-FLuc2 fusions in response to Nrf2-activators in cells (Nrf2-100-FLuc2, Nrf2-250-FLuc2, Nrf2-350-FLuc2). The activators used are DMSO, 5 μM PTS, 50 μM EGCG, 20 μM RES, 1 μM THA, 10 μM MMS, 10 μM DEM and 10 μM sodium arsenite. Asterisk (*) represents statistical significance (p<0.05) of signals compared to the ones obtained from DMSO treated cells. Error bars represent standard deviations (B) or SEM (C, D, E) of triplicate experiments.

Nrf2-FLuc2 fusion constructs expressing 50, 100, 150, 250, 350, and 435 N-terminal amino acids of Nrf2 protein were created **(Fig 2A)** under a constitutive expression system. The expression of Nrf2-FLuc2 fusion proteins by different constructs was confirmed by immunoblotting, and the results indicate step-wise increase in corresponding protein molecular weights, with FLuc-only plasmid transfected cells being at the bottom of the ladder, with subsequent increases corresponding to respective-length of Nrf2- protein fragment in each fusion construct **(Fig 2B)**. Although we were able to measure luciferase signal by luminometry assay, there is no visible band detected for fusion constructs expressing the 350 aa and 435 aa of Nrf2 protein, which may be a reflection of a high protein ubiquitinylation rate that makes them undetectable by immunoblotting. Nuclear translocation efficiency of abovementioned protein constructs was tested in MDA-MB231 cells transiently expressing each of the constructs to Nrf2 activators, e.g. NaAs, MMS, and DEM by assaying the luciferase activity **(Figure A in S1 File)**. Based on the preliminary screening, selective constructs that showed significant response to Nrf2 activators, such as Nrf2-100, 250 and 350, were further tested with a wider array of activators, e.g. PTS, EGCG, RES, THA, MMS, DEM and NaAs, and DMSO as solvent control **(Fig 2C–2E)**. Since Nrf2-100-FLuc2 construct demonstrated the strongest and most consistent level of activation in response to the abovementioned compounds, we then decided to focus our studies on this particular construct.

### Time and dose dependent response of Nrf2-100-FLuc2 construct to different Nrf2 activators

**Fig 3:**
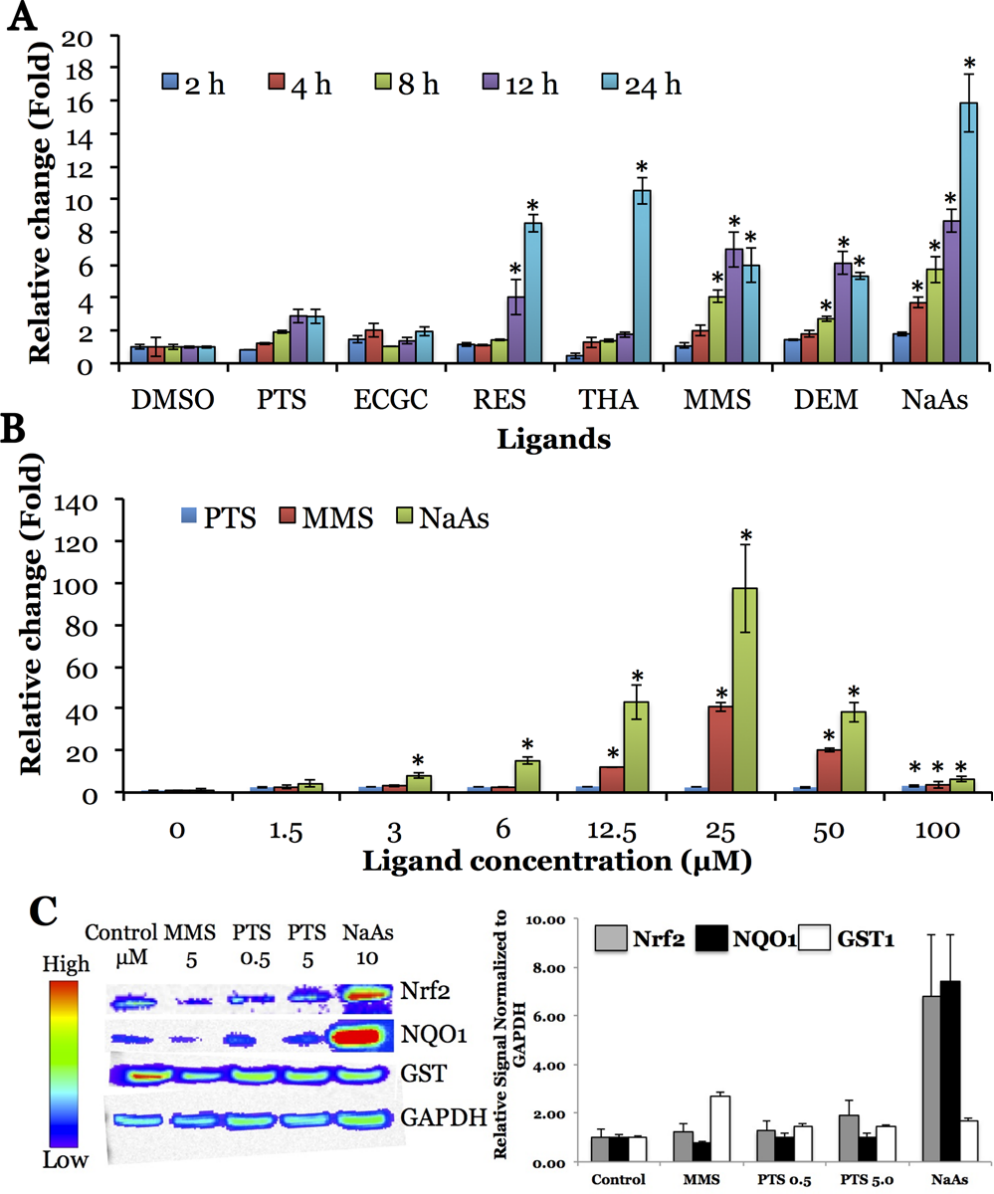
Time (A) and concentration (B) dependent activation of Nrf2-100-FLuc2 fusions in response to Nrf2-activators in cells. Asterisk (*) denotes statistical significance (p<0.05) compared to DMSO control (A) and non-exposed cells control (B). Expression of antioxidant genes in response to Nrf2 activators in MDA-MB231 cells stably expressing Nrf2-100aa-FLuc2 fusion. C, Immunoblot analysis of Nrf2, NQO1 and GST expression normalized to GAPDH expression and to control sample. The images were acquired by optical CCD (IVIS) camera imaging. Error bars represent SEM (A, B) or standard deviations (C) of triplicate experiments.

The construct expressing Nrf2-FLuc2 fusion protein with N-terminal Nrf2 of 100 aa was found to be significant enough in effectively inducing nuclear translocation of Nrf2-FLuc2 fusion protein, as judged by luciferase activity (50 fold basal level of activity, 4±2 fold as compared to DMSO carrier control). We used this particular construct for all further studies. Time and dose response experiments with respect to the exposure of different Nrf2 activators were carried out. The exposure times varied from 2 to 24 hours. Most Nrf2 activators showed maximum activity at 8-12h after exposure to the activators, thus it was found to be an optimal time point with respect to cell viability and increase in response (up to 6 fold relative to baseline); sodium arsenite (NaAs) showed maximum activity at 24 hours (∼16 fold increase relative to baseline) **(Fig 3A)**. Of the eight activators tested, MMS, PTS and NaAs were chosen for determining optimal concentration needed for efficient Nrf2 activation. Linear response to these activators’ concentrations was observed, with maximum activity reached at 25 μM (between 40 and 100 fold increase relative to baseline), with subsequent decline upon exposure to higher concentrations due to drug toxicity **(Fig 3B)**.

### Immunofluorescence analysis confirms the nuclear translocation of Nrf2-100-Luciferase in response to Nrf2 activator in cells

We have also conducted the immunofluorescence experiments, which indicate that Nrf2-100-Fluc2 fusion protein translocates into the nucleus when MDA MB231 cells were transfected and treated with Nrf2 activator PTS. The cells transfected with Nrf2-100-Fluc2 without PTS treatment, as well as cells transiently transfected with Fluc2 plasmid with and without PTS treatment served as experimental controls, and the results found no such translocation **(Figure B in S1 File)**.

### Evaluation of physical interaction of Nrf2-100-Fluc and Keap1 in MDA-MB231 cells transiently transfected with Nrf2-100-Fluc2 plasmid by immunoprecipitation

MDA-MB231 cells transiently transfected with the Nrf2-100-FLuc2 fusion reporter construct was used to analyze the interaction of this short Nrf2 (100 aa)-Luciferase fusion protein with the Nrf2 repressor molecule Keap1. The result of co-immunoprecipitation study using anti-Luciferase antibody for cell lysate pull-down, followed by the detection by anti-Keap1 antibody in immunoblot analysis, demonstrates that there is a strong interaction between the two molecules. The result is corroborated by the fact that when Nrf2 activator PTS was used for the treatment of the cells prior to lysis, the interaction between these two molecules was not observed, since PTS promoted Nrf2-100-Fluc2 translocation into the nucleus thereby making it inaccessible for the Keap1 protein. We did not observe any Keap1 band in cells transfected with FLuc2 construct and detected for Keap1 after pull-down by anti-Luciferase antibody from the lysates of cells treated with and without PTS further confirm the specificity of Nrf2-100-Luc2 interaction with Keap1 **(S2 File)**.

### Immunoblot analysis to confirm the Nrf2-activators mediated downstream target gene activation in cells

The expression of GST1 and NQO1, the downstream targets of Nrf2 was higher upon exposure of MDA-MB231 cells stably expressing Nrf2-100-FLuc2 to MMS, NaAs and PTS, while the expression of Nrf2 slightly increased compared to baseline, as determined by immunoblot analysis **(Fig 3C)**. The exposure of cells to NaAs showed consistently high level of expression of both Nrf2 and NQO1 while showing moderate increase in GST1 level.

### Evaluation of antioxidant chemosensitization in breast cancer cells

MDA-MB231 cells was used to analyze its response to cisplatin and 5-FU, the two clinically relevant anticancer drugs, and RRx-001, an epigenetically active drug, currently in a phase I clinical trial, when treated in combination with antioxidants EGCG or PTS. We also tested the effects of these combinational treatments on another breast cancer cell line (4T1-mouse mammary carcinoma), and a normal human breast epithelial cell line (Mcf10a).

#### Cytotoxicity evaluation of antioxidant EGCG and PTS in breast cancer cells

As the ultimate aim of this study is to evaluate the antioxidant mediated Nrf2 activation and chemosensitization in cancer therapy, first, we tested the apoptotic effects of two selective antioxidants applied alone at different concentrations and for various time points in MDA-MB231 triple negative human breast cancer cells, and 4T1 murine mammary adenocarcinoma cells. Neither of them demonstrated any significant apoptotic effect, with EGCG causing the maximum effect of 8.31, 13.3 and 15.6% at 48, 72 and 96 hours, respectively, when the highest concentration (50 μM) of EGCG was used **(Figure A in S3 File)**. The maximum apoptotic effect with the highest concentration of PTS (10 μM) used was 9.88% at 96 hours post treatment **(Figure B in S3 File)**. In contrast, both EGCG and PTS significantly enhanced the apoptotic rates in 4T1 cells when treated at the similar concentrations used for MDA-MB231 cells. Exposure to 50 μM of EGCG caused 38.2, 82.6, and 72% of apoptosis at 48, 72, and 96 hours, respectively **(Figure C in S3 File)** while PTS showing 37.4, 46.2 and 56.5% of apoptosis at similar time points **(Figure D in S3 File)** with 10 μM of PTS was used for the study.

#### Evaluation of antioxidant mediated chemosensitization of MDA-MB231 triple negative breast cancer cells to anticancer drugs

**Fig 4:**
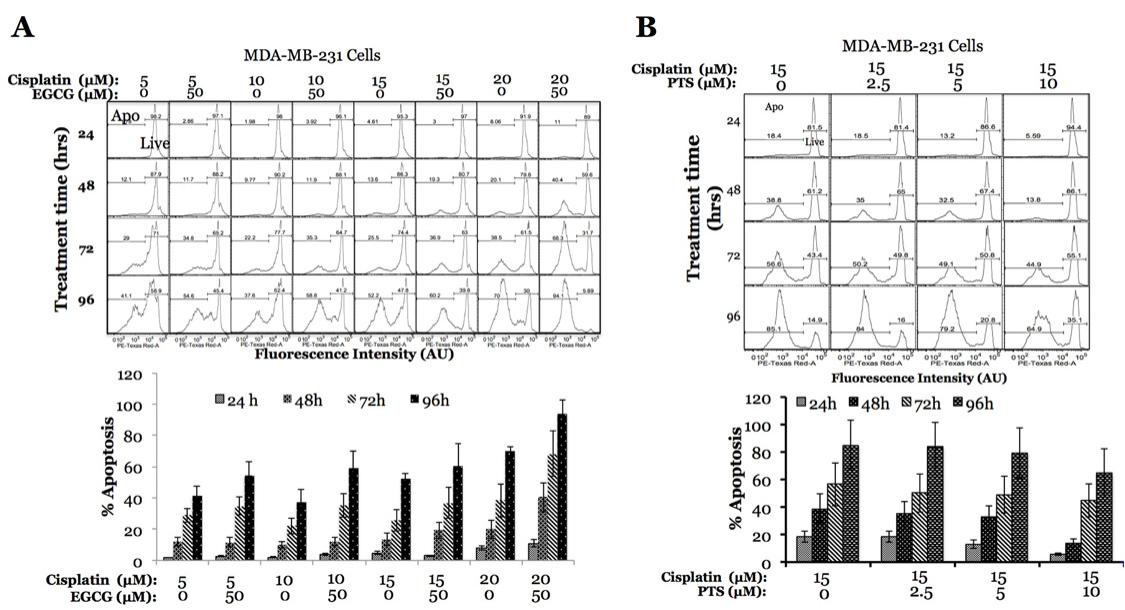
Effect of anticancer drugs in response to antioxidant-Nrf2-activators in MDA-MB231 Cells. A, Apoptotic effect of anticancer drug cisplatin (5–20 μM) in response to antioxidant EGCG (50 μM). B, Apoptotic effect of anticancer drug cisplatin (15 μM) in response to antioxidant PTS (0–10 μM. Error bars represent standard deviations of triplicate experiments.)

Since PTS and EGCG showed no toxicity in MDA-MB231 triple negative breast cancer cells at 10, 25 and 50 μM concentration while significantly enhancing the basal level of apoptosis in 4T1 cells, we evaluated the role of these antioxidants in improving the effect of chemotherapeutic drugs routinely used in the clinic. We used cisplatin at 5, 10, 15 and 20 μM concentrations in combination with 50 μM EGCG in MDA-MB231 cells, and compared treatment results with the cells treated with drug alone. The increase of apoptosis rate was noticeable at 72 hours from treatment initiation for 5 and 10 μM cisplatin concentrations (15% on average), and became significant at 20 μM cisplatin concentration as early as 48 hours after treatment initiation (40.4 vs 20.1%), continuing to increase at 72 (38.5 vs 68.3%) and 96 hours (94.1 vs 70%) post treatment initiation **(Fig 4A)**. In contrast to chemosensitization effect observed from EGCG, the co-treatment of MDA-MB231 cells with antioxidant PTS with cisplatin significantly stabilized the effect of cisplatin. We observed a reduction in apoptotic rates of cisplatin alone treatment (15 μM), which dropped from 18.4 to 5.59%, 38.8 to 13.8%, 56.6 to 44.9% and 85.1% t0 64.9% when co-treated with PTS, at 24, 48, and 72 hours, respectively **(Fig 4B)**.

Since we observed significant chemosensitization from EGCG when treated with cisplatin, we further tested EGCG with another commonly used chemotherapeutic agent 5-Fluorouracil (5-FU) in MDA-MB231 cells. Apoptosis rate increased significantly at 48 and 72 hours post-treatment initiation when cells were treated with 50 μM of 5-FU in combination with 50 μM EGCG as compared with drug alone treatment (17.1 vs 7.85% and 42.9 vs 32.4%, at 48 and 72 hours, correspondingly), but observed a dose dependent drop at 96 hours (55.7 and 47.6 at 25 and 50 μM EGCG vs 59.4 with drug alone treatment), implicating EGCG’s protective action with respect to 5-FU treatment at later time points **(S4 Flie).**

#### Evaluation of antioxidant chemosensitization mediated apoptotic induction by RRx-001 in MDA-MB231 breast cancer cells

**Fig 5:**
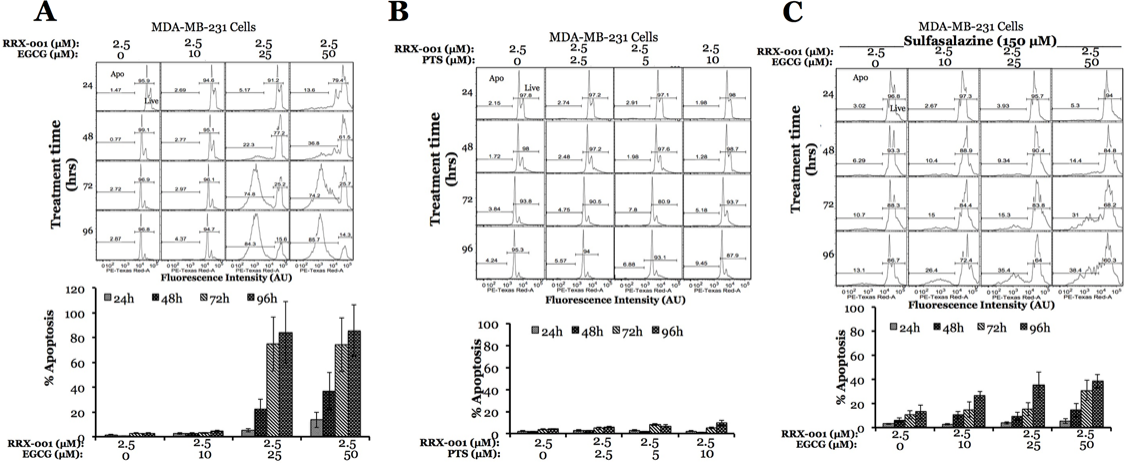
Effect of anticancer drugs in response to antioxidant-Nrf2-activators in MDA-MB231 cells. Apoptotic effect of anticancer drug RRx-001 (2.5 μM) in response to antioxidants EGCG (0–50 μM) (A) and PTS (0–10 μM) (B). C, Effect of GSH pathway inhibitor sulfasalazine on apoptotic rates of cells treated with anticancer drug RRx-001 (2.5 μM) with or without EGCG (0–50 μM). Error bars represent standard deviations of triplicate experiments.

After the evaluation of two antioxidants (PTS and EGCG) with two commonly used chemotherapeutic agents (Cisplatin and 5-FU), we tested another chemotherapeutic drug, RRx-001, currently in phase I clinical trial for the treatment of lymphoma[16], in combination with PTS and EGCG. Most dramatic effect of drug-antioxidant treatment was observed when the MDA-MB231 cells were treated with 2.5 μM of RRx-001 in combination with increasing concentrations of EGCG (10, 25 and 50 μM). Treatment with drug alone produced almost no effect as far as apoptosis rate is concerned, while it increased to nearly 6 and 13% in 24 hours, 23 and 37% in 48 hours, 75% in 72 hours and 85 and 86% in 96 hours when co-treated with 25 and 50 μM EGCG, respectively **(Fig 5A)**, while PTS didn’t show any apoptotic effect, being constant around 5% for all conditions and at all time points studied **(Fig 5B)**.

We then explored a possibility of a pathway other than Nrf2 being involved in the antioxidant response, such as GST pathway. For that, we tested MDA-MB231 cells in the presence of anticancer drug RRx-001 in the presence of antioxidant EGCG by co-treating with anti-inflammatory drug sulfasalazine, which has previously been shown to block xCT-symporter that is important for the exchange of cystine and glutamate across cell membrane during GSH synthesis[17]. The intention was to further improve the effect of antioxidant-chemotherapy combination. In contrast, we observed significant reduction in the apoptotic effect when exposed cells to 150 μM of sulfasalazine before treating with EGCG and RRx-001 combinations. The results of apoptosis induced by EGCG and RRx-001 combinations when pretreated with 150 μM of sulfasalazine dropped to more than two-fold when highest concentration of EGCG (50μM) of used in combination with RRx-001 (5.3 *vs* 13.6%, 14.4 *vs* 36.8%, 31 *vs* 74.2% and 38.4 *vs* 85.7% at 24, 48, 72 and 96 hours, correspondingly) **(Fig 5A and 5C)**.

#### Evaluation of RES and MMS mediated chemosensitization of MDA-MB231 breast cancer cells to cisplatin

We also evaluated to additional antioxidants, such as RES and MMS for their ability in chemosensitizing MDA-MB231 cells to cisplatin by co-treating with different combinations. While 10 μM RES alone induced 41% of the cells to undergo apoptosis, as low as 2.5 μM RES, resulted in 26% of cell death when applied in combination with 15 μM cisplatin **(Figure A in S5 File)**. Not so dramatic, though, significant effect was observed when the same cells were treated with 2.5 to 10 μM of MMS in combination with 15 μM cisplatin. MMS alone did not show significant apoptotic effect at these concentrations **(Figure B in S5 File)**.

To examine the effect of anticancer drug RRx-001 in the presence of antioxidants on another phenotypically different breast cancer cells and in normal cells, we conducted analogous experiments in 4T1-murine breast cancer cells and MCF10a-normal breast epithelial cell line. As mentioned above, antioxidants alone significantly increased apoptosis in 4T1 cells, while no such effect was observed in MDA-MB231 cells. 4T1 cells are also more sensitive than MDA-MB231 cells to RRx-001 drug alone treatment, and when the treatment was combined with antioxidants, they demonstrated a very high apoptotic rates as early as 48 hours post treatment initiation **(Figures A and B in S6 File)**. Importantly, EGCG treatment had no apoptotic effect on non-cancerous MCF10a breast epithelial cells, and the basal rate of apoptosis was similar across the board for RRx-001 and EGCG combinations at all time points studied **(Figures A and B in S7 File)**. These results demonstrated that administering antioxidants along with anticancer therapy may significantly improve therapeutic outcome in that smaller dose, and even may require shorter treatment times to achieve the desired effects, i.e. cancer cell apoptosis, while not affecting normal cells. However, the antioxidant mediated chemosensitization strategy cannot be generalized for any given cancer cell type, requiring separate evaluation for every type of chemotherapeutic drug/antioxidant combination.

To further decipher the role of Nrf2 in chemosensitization, we used siRNA transfection to downregulate endogenous Nrf2 level in MDA-MB231 cells, and exposed them to 10 μM EGCG, 15 μM cisplatin, and combination of both for 48 hours. The cells transfected with scrambled (Sc) siRNA and treated at similar concentration of drugs served as matched controls. We observed a significant increase in apoptotic rate in cells transfected with Nrf2 specific siRNA when compared to scrambled siRNA in all treatment conditions **(Figure A in S8 File)**. The immunoblot analysis showed significant downregulation of endogenous Nrf2 level in cell transfected with Nrf2-siRNA compared to Sc-siRNA transfected cells **(Figure B in S8 File)**.

### Evaluation of antioxidant chemosensitization mediated activation of ARE-signaling by RRx-001 in MDA-MB231 breast cancer cells by Nrf2-100-FLuc2 reporter system

**Fig 6:**
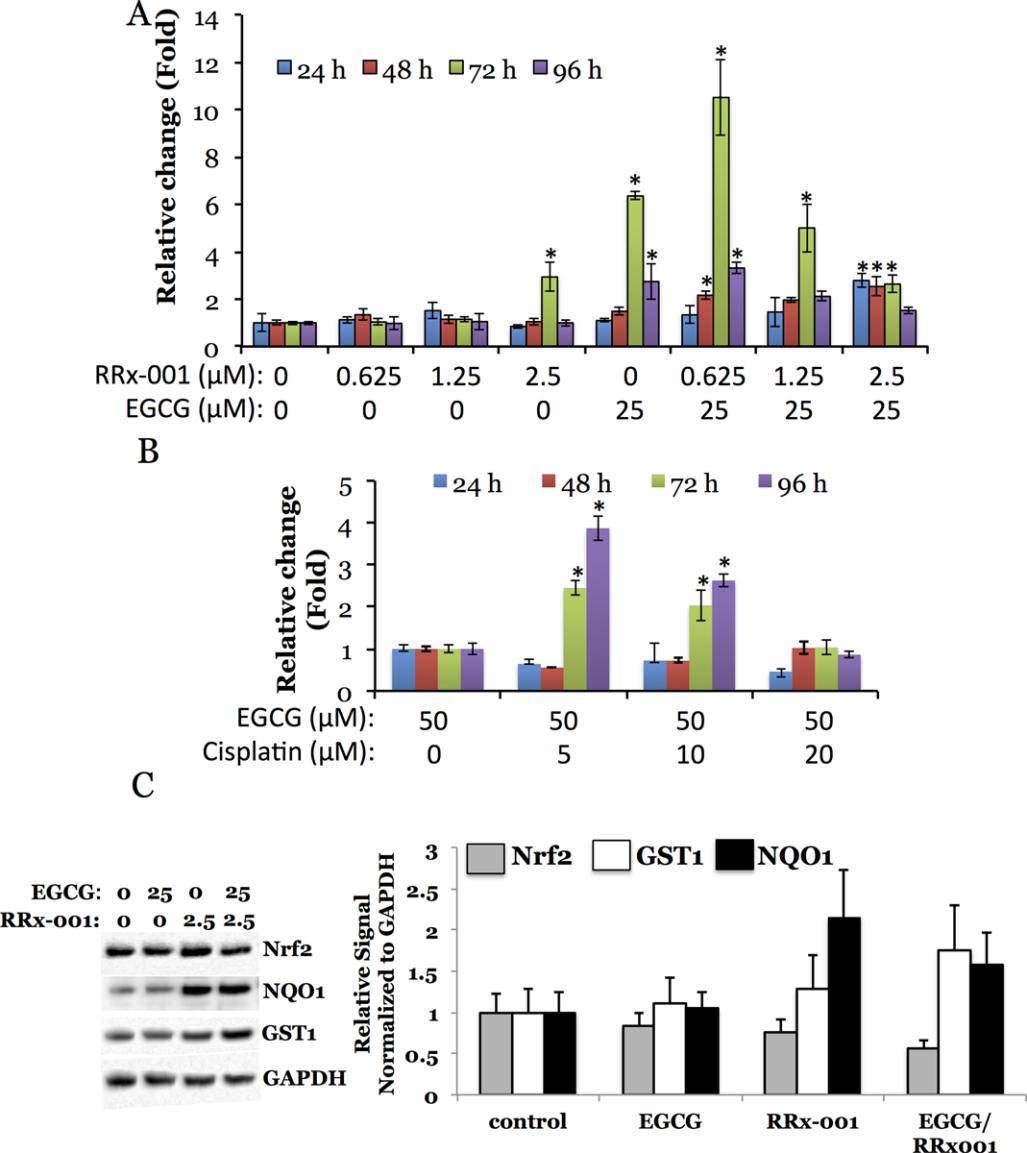
Time dependent activation of Nrf2-100-FLuc2 fusions in response to varying anticancer drug (A, RRx-001 and B, cisplatin) concentration in the presence of antioxidant (EGCG) in MDA-MB231 cells, and Nrf2 target genes (GST and NQO1) expression in MDA-MB231-Nrf2-100-FLuc2 cells in response to anticancer drug (RRx-001) in the presence of antioxidant (EGCG). Asterisk (*) denotes statistical significance (p<0.05) of a signal compared to that from the untreated cells (A) and from cells treated with 50 μM EGCG alone (B). C, Immunoblot of cell lysates treated with drug and/or antioxidant for 24 hours (Nrf2, GST and NQO1 expression was normalized to GAPDH expression). Error bars represent standard deviations of triplicate experiments.

To evaluate the efficiency of Nrf2 activation in response to the treatment of a combination of antioxidant (EGCG) and chemotherapeutic drug combination (RRx-001 or Cisplatin), we used MDA-MB231 cells stably expressing Nrf2-100-FLuc2 reporter protein. The cells were measured for luciferase activity different time points after treatment. We found that even a drug concentration below its IC50 dose showed significant level of luciferase activation when combined with antioxidant. The drug RRx-001 resulted in nearly 12 fold activation at 72 hours post-treatment when combined with EGCG, as compared drug alone treatment and to baseline no treatment control. Similarly, cisplatin showed 3 to 4-fold activation when combined with EGCG **(Fig 6A and 6B)**. The immunoblot analysis of Nrf2 and its downstream target genes found that the GST1 and NQO1 proteins expression were increased in response to RRx-001 and EGCG combination treatment, as compared to control cells **(Fig 6C)**.

**Fig 7:**
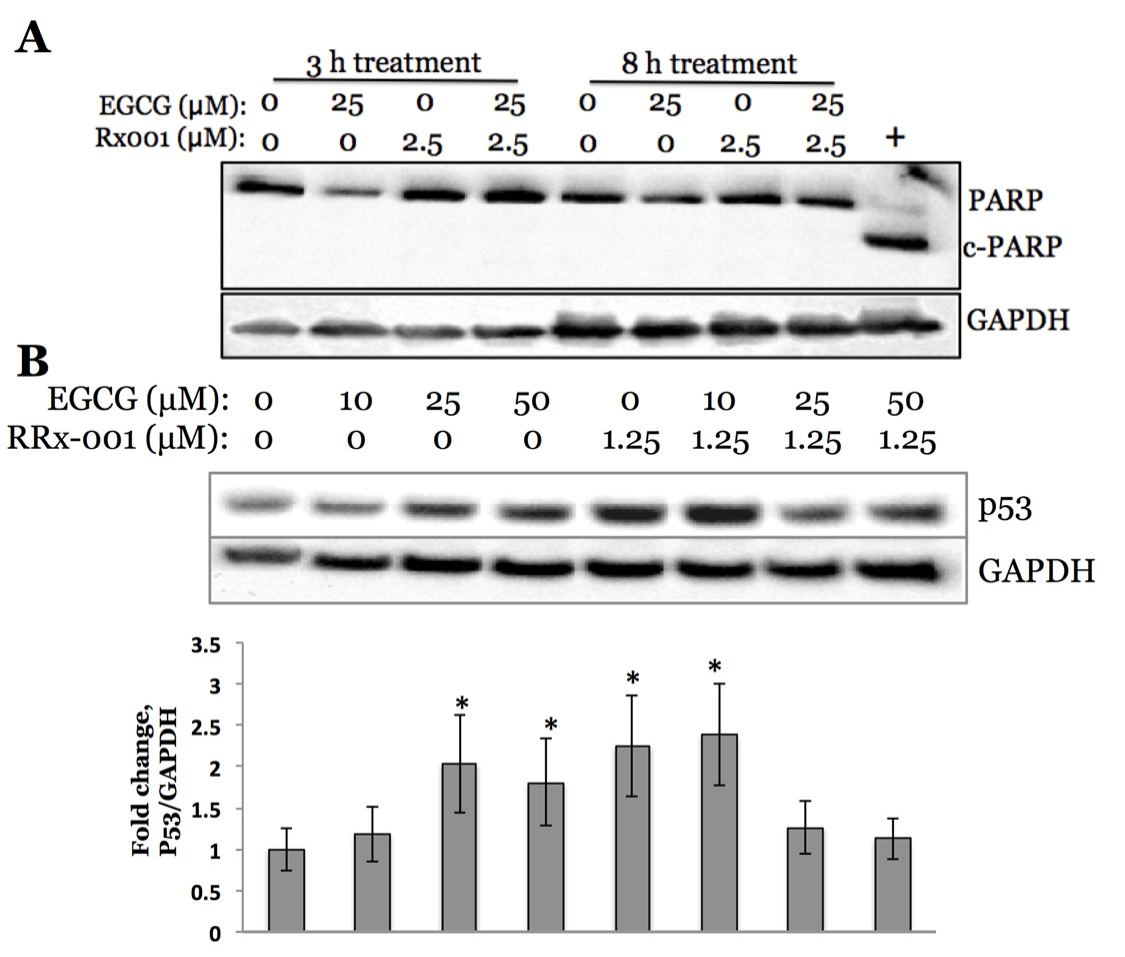
A, MDA-MB231 cells exposure to antioxidant activator of Nrf2 (EGCG, 25 μM), chemotherapeutic agent RRx-001 (2.5 μM) and combination treatment play no role in caspase pathway after 3 and 8 hours of exposure, evident from the absence of PARP inhibition as detected by immunoblot. Jurkat cells treated with 200 μM staurosporine for 3 hours served as positive control. **B, Immunoblot analysis of p53 expression in MDA-MB231 cells exposed to antioxidant activator of Nrf2 (EGCG, 25** μ**M), chemotherapeutic agent RRx-001 (1.25** μ**M) and combination treatment (48 hours post exposure).** Asterisk (*) denotes statistical significance (p<0.05) of signals compared to the ones obtained from untreated control cells. Error bars represent standard deviations of triplicate experiments.

To test the possible role of Nrf2 in caspase pathway, we exposed MDA-MB231 cells to EGCG, RRx-001 or EGCG/RRx-001 combination for 3 hours and 8 hours, in order to detect possible role of poly ADP ribose polymerase (PARP) cleavage, the downstream target of caspase pathway. For this, immunoblot analysis of corresponding cell lysates was performed **(Fig 7A)**. However, no effect on PARP cleavage was detected, as would have been evident from the appearance of a cleaved lower molecular weight (89kDa) band representative of cleaved protein, as opposed to a full-length (130kDa) protein in the untreated cell lysate lane. Jurkat cells treated with 200 μM of staurosporine for 3 hours served as positive control.

Finally, we have explored a status of p53 by exposing MDA-MB231 cells to EGCG, RRx-001 or EGCG/RRx-001 combination for 48 hours, and subjecting collected cell lysates to immunoblot analysis. Our results indicate that p53 level gradually increased when cells were exposed to 10, 25 and 50 μM EGCG alone, RRx-001 alone, and RRx-001 in combination with 10 μM of EGCG, leveling off when cells were exposed to RRx-001 in combination with 25 or 50 μM EGCG, as compared to untreated control cells **(Fig 7B)**.

### Combinatorial treatment evaluation in human tumor xenograft of MDA-MB231 cells in mouse

**Fig 8:**
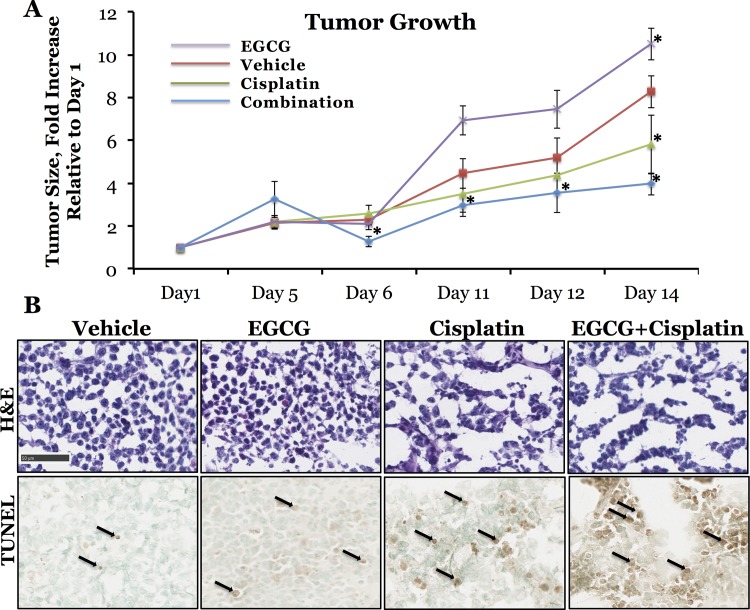
A, Rate of MDA-MB231 tumor xenografts growth in mice treated by vehicle control, cisplatin, EGCG and cisplatin/EGCG combination. Asterisk (*) denotes statistical significance of data obtained from mice treated with EGCG (Day 14, P = o.046), cisplatin (Day 14, p = 0.036) and combinational treatment (Day 6, p = 0.048; Day 11, p = 0.014; Day 12, p = 0.019 and Day 14, p = 0.016) compared to vehicle control treated mice. Error bars represent SEM of multiple tumors. **B, *Ex-vivo* analysis of MDA-MB231 tumor xenografts**. H&E staining, top row, TUNEL staining for apoptotic cells, bottom row. Arrows represent nuclear TUNEL staining.

Four groups of mice (n = 5) implanted with TNBC tumor xenografts were subjected to different treatment regimens, after tumor volumes reached approximately 100–200 mm^3^, when treatment was initiated (Day 0). Four different treatment conditions included vehicle control, cisplatin alone, EGCG alone and cisplatin/EGCG combination treatment. The mice were treated on Day 0, 5, and 11, and tumors were measured on Days 0, 1, 5, 6, 11, 12 and 14. The results indicate that in the group of mice treated with cisplatin/EGCG combination, tumor size increase relative to Day 0 (before treatment initiation) was much lower (3.96 ± 0.51 fold) than in mice treated with vehicle carrier (8.27 ±0.74 fold) and EGCG alone (10.51 ± 3.11 fold), and, most importantly, it was lower than in mice treated with cisplatin alone (5.81 ± 1.38 fold) (**Fig 8A**). These results support our claim that the combination treatment exerts synergistic effect on triple negative breast tumor growth, supporting our *in vitro* results. Upon an *ex-vivo* analysis of tumors, we observed a much higher amount of apoptotic cells in the tumors extracted from the mice treated with a combination of cisplatin and EGCG, as compared to those extracted from mice treated with cisplatin alone; only a basal level of apoptosis was observed in tumors obtained from the mice treated with EGCG alone or with vehicle control (**Fig 8B**).

## Discussion

In this study, we have developed a reporter system that measures the activation Nrf2 in response to its activators without affecting endogenous target genes expressions. The system was used to study the role of Nrf2 activators in cancer cell sensitization during chemotherapy. N-terminal 435-amino acids of Nrf2 are assumed to be crucial for its binding to Keap1 during its ubiquitination, but the exact binding site or the minimum N-terminal Nrf2 domain required for efficient Keap1 binding without altering endogenous ARE-signaling is unknown. We constructed luciferase fusion with N-terminal Nrf2 fragments of different amino acid lengths (50, 100, 150, 200, 250, 350, and 435 aa) and examined the efficiency of luciferase activation in response to Nrf2 activators in the presence and absence of anticancer drugs in MDA-MB231 triple negative breast cancer cells. As evident from the luciferase assay that served as a direct measurement of Nrf2 translocation into the nucleus, the construct expressing Nrf2-FLuc2 fusion protein with N-terminal Nrf2 of 100 aa was found to be significant enough for efficient translocation. The luciferase activity increased 50 fold upon exposure of cells to Nrf2 activators, and 4±2 fold as compared to DMSO carrier control. Of the eight activators tested, MMS, PTS and NaAs were found the most effective in activating ARE signaling. Linear response to these activators’ concentration was observed, with maximum activity reached at 25 μM, and subsequent decline upon exposure to higher concentrations, mainly due to drug cytotoxicity. We compared the activation response of cells expressing Nrf2-100-FLuc2 to those expressing hNQO1-ARE-Luc and GST-ARE-Luciferase gene constructs that are considered “gold standards” for studying ARE pathway. Nrf2-100-FLuc2 constructs expressing cells were found to be many-fold more efficient in response to ARE pathway activators than the other two, which indicates that Nrf2-100-FLuc2 construct is a valuable tool for studying ARE-signaling pathway and evaluation of its activators (data not shown). The expression of GST and NQO1, the downstream targets of Nrf2 was higher upon exposure to MMS, PTS and NaAs, in accordance with increase in luciferase signal.

Since bioluminescence imaging can be used to monitor various biological pathways in living animals by non-invasive imaging, the optimal Nrf2-100-FLuc2 fusion construct developed in this study can be a valuable tool for monitoring anticancer therapy effect in various tumors by using tumor cell xenografts expressing Nrf2-100-FLuc2 construct to study the role of Nrf2-ARE signaling in cancer therapy and drug resistance, without supplementing functional effect, which in our opinion, constitutes the novelty of this study.

One of the strategies to overcome chemoresistance is chemosensitization. The mechanisms of chemoresistance in tumor cells may be intrinsic or acquired. They include alterations of drug influx or efflux [18], inactivation of chemotherapeutic agents through metabolism [19], enhanced DNA repair [20], altered cell death regulation [21], and many more. Chemosensitization may provide a way to overcome chemoresistance by affecting one or more of these mechanisms, and antioxidants have been reported to play a role of sensitizers. For example, *in vitro* and *in vivo* studies have shown that resveratrol (RES) serves as a sensitizer by modulating apoptotic pathways, down-regulating drug transporters, and down-regulating proteins involved in tumor cell proliferation [14], and our results obtained when cells were treated with RES in combination with cisplatin support this result. To address the possibility of chemosensitization in MDA-MB231 triple negative breast cancer cells, MDA-MB231 cells were analyzed for their response to conventional anticancer drugs (cisplatin, 5-FU) and a drug currently in clinical trial (RRx-001) by combining with antioxidants EGCG or PTS.

For example, when these cells were treated with cisplatin (5–20 μM concentrations) in combination with 50 μM EGCG, as compared to treatment with drug alone, the overall increase of apoptosis rate was noticeable at all time points examined (between 24 and 96 hours post treatment initiation) and at wide range of cisplatin concentrations. In contrary, when cells were treated with 50 μM of 5-FU in combination with 50 μM EGCG as compared with drug alone treatment, we have observed a decrease in apoptosis at later time points, followed initial increase, implicating the protective role of EGCG with respect to 5-FU treatment **(S3 File)**. Similar to this, increasing concentrations of PTS demonstrated the same protective action with respect to cisplatin treatment at earlier time points **(Fig 4B)**. Co-treatment with EGCG in combination with chemotherapeutic drug RRx-001 demonstrated advantageous effect as far as very significant increase in apoptosis as early as 24 hours post-treatment initiation, while PTS protected cancer cells when combined with RRx-001, similar to cisplatin. These differential results further highlight the necessity of understanding the combinational treatment application timeline and fine-tuning the concentrations of drug/antioxidants combinations. Keeping in mind that MDA-MB231 cell line is one of the most resistant cells to chemotherapy, we tested the same combinations in other cell lines, such as 4T1 and MCF10a. Interestingly, antioxidants EGCG and PTS alone promote 50–75% of the 4T1 cells to apoptosis at a later time point and at concentrations that showed no effect on MDA-MB231 cells. Notably, 4T1 cells were also more sensitive than MDA-MB231 cells to RRx-001 drug treatment alone, with no addition of EGCG or PTS, and when the treatment was combined with these antioxidants, they demonstrated very high apoptotic rates at early times post treatment initiation. One of the important findings of this study is establishing the difference between the two breast cancer cell lines in their response to treatments, which demonstrates that antioxidant mediated chemosensitization concept cannot be generalized for any given cancer type, requiring separate evaluation for every type of chemotherapeutic drug/antioxidant combination. To our knowledge, there are no comprehensive reports demonstrating the importance of this phenomenon. Importantly, EGCG treatment had no effect on apoptosis rate in non-cancerous Mcf10a breast epithelial cell line, and apoptosis was similar across the board for RRx-001/EGCG combinations at all time points, 25% on average regardless of EGCG concentration, which is an encouraging result in that it alleviates the concern of normal cells being affected by antioxidant treatment.

To evaluate the efficiency of Nrf2 activation indirectly in cells, we conducted luciferase assay experiments with Nrf2-100-FLuc2 expressing MDA-MB231 cells that were grown in the presence of chemotherapeutic drugs RRx-001 or cisplatin alone, or in combination with antioxidant EGCG. We consider of special importance the results demonstrating that even a smaller drug dose administered in combination with this Nrf2 activator resulted in a more efficient Nrf2 activation than a higher dose of chemotherapeutic agents administered alone, which is holding a potential when considering translation of these results into the clinical setting.

We also tested the possible pathways involved in the antioxidant response induced apoptosis, such as GST, p53, PARP, and caspase pathways. When MDA-MB231 cells were grown in the presence of anticancer drug RRx-001 with or without antioxidant EGCG in the presence of sulfasalazine, an anti-inflammatory drug which is known for inhibiting xCT-symporter involved in cystine/glutamate exchange during GSH synthesis, apoptotic response of these cells showed no difference when compared to that of the cells grown at the same drug/antioxidant conditions without sulfasalazine [22]. While supplementing sulfasalazine is expected to induce apoptosis, the results of this study show significant reduction in the apoptotic effect in cells co-treated with RRx-001 and EGCG. This is an important observation, since one of the chemoresistance mechanisms in human breast cancer cells has been shown to be mediated through glutathione/glutathione S-transferase system, and there are reports showing that resistance to chemotherapeutic drugs is associated with increase in the concentration of GSH, or expression of GST [23, 24].

To test the possible role of Nrf2 in caspase pathway, we have exposed MDA-MB231 cells to EGCG, RRx-001 or EGCG/RRx-001 combination, in order to detect possible cleavage of poly ADP ribose polymerase (PARP), the downstream target of caspase pathway, by abovementioned compounds. Poly(ADP-ribose) polymerase (PARP), a nuclear protein implicated in DNA repair, is one of the earliest proteins targeted for a specific cleavage to the signature 89kDa fragment during apoptosis. Characterization of the apoptotic cleavage of PARP and other target proteins helped in understanding the role of cysteine aspartic acid specific proteases (caspases) in the apoptotic process [25]. There is a report indicating that increased levels of PARP were found in cisplatin-resistant head and neck tumor cell line [26]. Our results indicate that there is no observed effect on PARP cleavage upon applying combination therapy to MDA-MB231 cells, which allows for a conclusion that caspase and Nrf2 pathways do not overlap, especially concerning the combination of antioxidant and chemotherapeutic drug used for the study (EGCG with RRx-001).

Another mechanism by which tumor cells become resistant to chemotherapeutic agents is by acquisition of resistance to cell death mechanisms, specifically to apoptosis. One of the ways by which it happens is by down-regulating pro-apoptotic proteins, e.g. p53 [21, 27]. In regard to antioxidants, recent report by Sayin *et al*. indicates that NAC and vitamin E increase tumor cell proliferation by reducing ROS, DNA damage, and p53 expression in mouse and human lung tumor cells and that inactivation of p53 increases tumor growth to a similar degree as antioxidants and abolishes the antioxidant effect [28]. Our investigation demonstrated the opposite effect, in that p53 levels marginally increased with increasing antioxidant (EGCG) concentration, as well as when combinational treatment was applied to triple negative breast cancer cells.

Most importantly, our *in-vitro* developed hypothesis was corroborated by our *in vivo* results indicating suppression of tumor growth and increased apoptotic response in tumors of animals that received combinational cisplatin/EGCG therapy. The cytoprotective attributes of the Nrf2 signaling pathway have been targeted for chemoprevention, as administration of Nrf2-inducing agents has been shown to result in decreased carcinogenesis in animal models and altered carcinogen metabolism in humans. On the other hand, decreased expression or altered function of the enzymes that are part of the Nrf2 signaling pathway can lead to differential susceptibility to disease [29] while mutations in the Nrf2 signaling pathway have been shown to be an effective mechanism for cancer cells to evade chemotherapy. Overall, our study supports the claim that the Nrf2 cytoprotective adaptive response has evolved to be a powerful protective strategy for organisms against exposure to environmental toxicants and may provide insight into differential disease susceptibilities across populations and responses to therapies designed to alleviate these conditions.

## Supporting Information

S1 File Preliminary evaluation of all Nrf2-Luciferase (Nrf2-Fluc2) truncation constructs in 293T transiently transfected cells (Figure A in S1 File). **Nuclear translocation of Nrf2-100-Fluc2 fusion construct introduced by transient transfection into MDA-MB231 cells in response to 12 h treatment by 5** μ**M PTS (right panel-top) (Figure B in S1 File).**
(PDF)

S2 File Evaluation of physical interaction of Nrf2-100-Fluc2 and Keap1 in MDA-MB231 cells transiently transfected with Nrf2-100-Fluc2 plasmid by immunoprecipitation. (PDF)

S3 File Concentration and time-dependent effect of antioxidant EGCG on MDA-MB231 cells (Figure A in S1 File). Concentration and time-dependent effect of antioxidant PTS on MDA-MB231 cells (Figure B in S1 File). Concentration and time-dependent effect of antioxidant EGCG on 4T1 cells (Figure C in S1 File). Concentration and time-dependent effect of antioxidant PTS on 4T1 cells (Figure D in S1 File).
(PDF)

S4 File Apoptotic effect of anticancer drug 5-Fluorouracil (50 μM) in response to antioxidant EGCG (0–50 μM) on MDA-MB231 cells. (PDF)

S5 File Apoptotic effect of antioxidant RES (0–10 μM) in the presence or absence of anticancer drug cisplatin (15 μM) in MDA MB231 cells (A). **Apoptotic effect of antioxidant MMS (0–10** μ**M) in the presence or absence of anticancer drug cisplatin (15** μ**M) in MDA MB231 cells (Figure B in S1 File).**
(PDF)

S6 File Apoptotic effect of anticancer drug RRx-001 (2.5 μM) in response to antioxidant EGCG (0–50 μM) on 4T1 cells (Figure A in S1 File). **Apoptotic effect of anticancer drug RRx-001 (2.5** μ**M) in response to antioxidant PTS (0–25** μ**M) on 4T1 cells (Figure B in S1 File).**
(PDF)

S7 File Apoptotic effect of antioxidant EGCG (0–50 μM) in Mcf10a cells (Figure A in S1 File). **Effect of anticancer drug RRx-001 in response to antioxidant-Nrf2-activator EGCG in Mcf10a cells in combination with antioxidant EGCG (0–50** μ**M) (Figure B in S1 File).**
(PDF)

S8 File Apoptotic effect of antioxidant EGCG (10 uM) in the presence and absence of chemotherapeutic drug cisplatin (15 μM) in MDA MB231 cells treated with Nrf2-specific or scrambled siRNA (Figure A in S1 File). **Immunoblot analysis demonstrating Nrf2 expression in MDAMB231 cells treated with scrambled and Nrf2-specific siRNAs.** Quantitative plot is shown on the right; error bars represent standard deviations of triplicate experiments **(Figure B in S1 File).**
(PDF)
