# Supplementary material for: Monitoring the Antioxidant Mediated Chemosensitization and ARE-Signaling in Triple Negative Breast Cancer Therapy
Source: PLoS One. 2015 Nov 4;10(11):e0141913. doi: 10.1371/journal.pone.0141913 (PMC4633093; doi:10.1371/journal.pone.0141913)
Supplement: S1 File — Nuclear translocation of Nrf2-100-Fluc2 fusion construct introduced by transient transfection into MDA-MB231 cells in response to 12 h treatment by 5 μM PTS (right panel-top) (Figure B in S1 File). (PDF) [file pone.0141913.s001.pdf]

Supplementary Information Foygel et al.

A

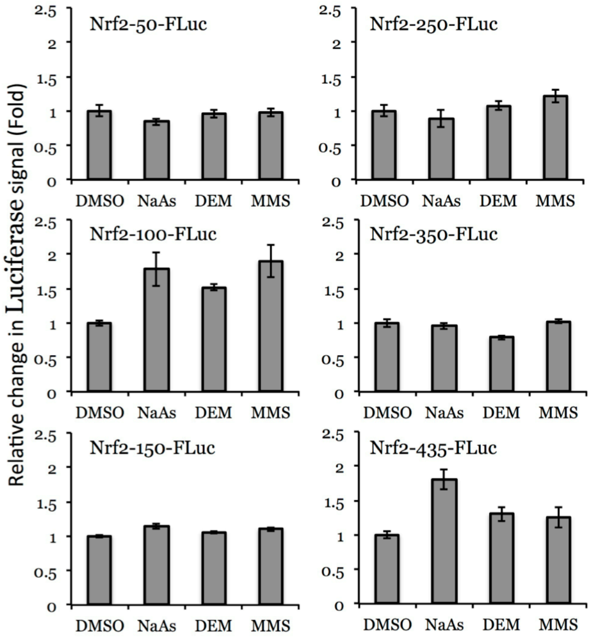

B

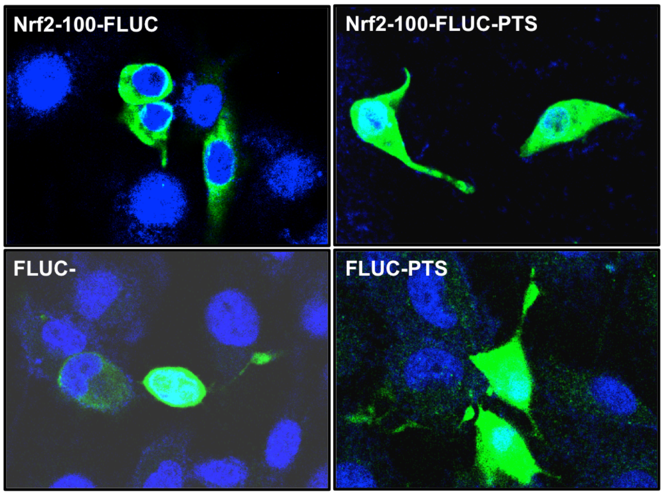

S1 File. Figure A. Preliminary evaluation of all Nrf2-Luciferase (Nrf2-Fluc2) truncation constructs in 293T transiently transfected cells. Cells were subjected to exposure to following Nrf2 activators: DMSO, 10 µM NaAs, 10 µM DEM and 10 µM MMS. Asterisks (*) denote statistical significance ($p<0.05$) of signal compared to one from DMSO control cells.

Figure B. Nuclear translocation of Nrf2-100-Fluc2 fusion construct introduced by transient transfection into MDA-MB231 cells in response to 12 h treatment by 5 µM PTS (right panel-top). Bottom left panel shows control cells untreated by PTS. Bottom panels represent images of cells transiently transfected with Fluc2 only plasmid, where Luciferase cellular location is independent of PTS treatment. Nuclei are stained blue (DAPI), Luciferase is stained green (anti-Luciferase antibody).
