# Supplementary material for: Monitoring the Antioxidant Mediated Chemosensitization and ARE-Signaling in Triple Negative Breast Cancer Therapy
Source: PLoS One. 2015 Nov 4;10(11):e0141913. doi: 10.1371/journal.pone.0141913 (PMC4633093; doi:10.1371/journal.pone.0141913)
Supplement: S2 File — (PDF) [file pone.0141913.s002.pdf]

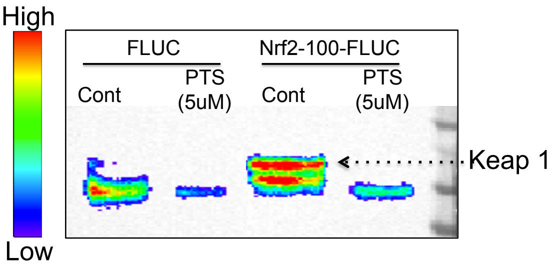

S2 File. Evaluation of physical interaction of Nrf2-100-Fluc2 and Keap1 in MDA-MB231 cells transiently transfected with Nrf2-100-Fluc2 plasmid by immunoprecipitation. The cell lysates were immunoprecipitated by FLuc antibody and detected using Keap1 antibody. Immunoblot analysis shows a significant Keap 1 band in cells transfected with Nrf2-100-Fluc2, and the band is lost in cells treated with PTS. The cells transfected with Fluc2 plasmid show no Keap 1 band in both conditions (with and without PTS)
