# Supplementary material for: Monitoring the Antioxidant Mediated Chemosensitization and ARE-Signaling in Triple Negative Breast Cancer Therapy
Source: PLoS One. 2015 Nov 4;10(11):e0141913. doi: 10.1371/journal.pone.0141913 (PMC4633093; doi:10.1371/journal.pone.0141913)
Supplement: S3 File — Concentration and time-dependent effect of antioxidant PTS on MDA-MB231 cells (Figure B in S1 File). Concentration and time-dependent effect of antioxidant EGCG on 4T1 cells (Figure C in S1 File). Concentration and time-dependent effect of antioxidant PTS on 4T1 cells (Figure D in S1 File). (PDF) [file pone.0141913.s003.pdf]

Supplementary Information Foygel et al.

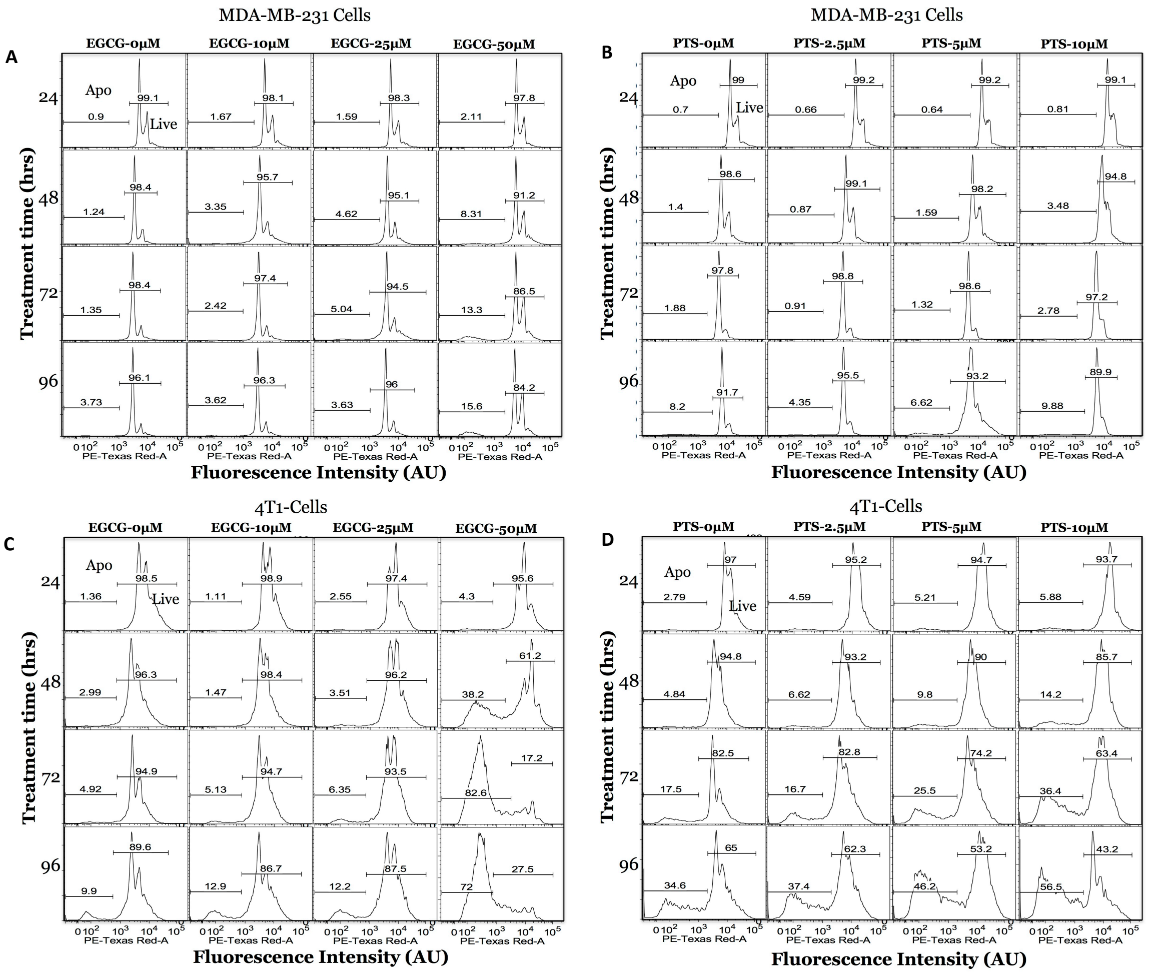

S3 File. Concentration and time-dependent effect of antioxidant EGCG (Figure A) and PTS (Figure B) on MDA-MB231 cells. Concentration and time-dependent effect of antioxidant EGCG (Figure C) and PTS (Figure D) on 4T1 cells.
