# Supplementary material for: Monitoring the Antioxidant Mediated Chemosensitization and ARE-Signaling in Triple Negative Breast Cancer Therapy
Source: PLoS One. 2015 Nov 4;10(11):e0141913. doi: 10.1371/journal.pone.0141913 (PMC4633093; doi:10.1371/journal.pone.0141913)
Supplement: S4 File — (PDF) [file pone.0141913.s004.pdf]

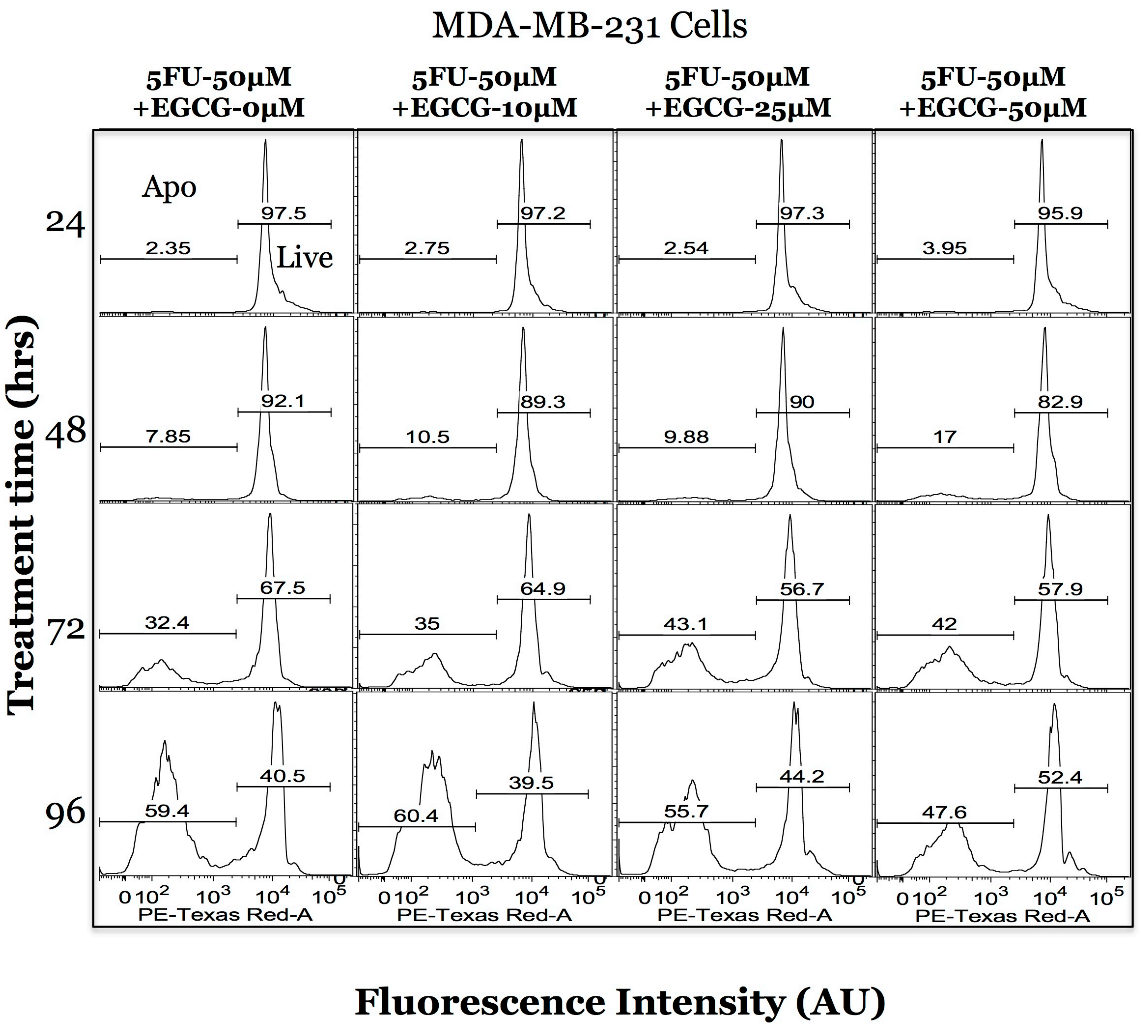

S4 File. Apoptotic effect of anticancer drug 5-Fluorouracil (50 µM) in response to antioxidant EGCG (0-50 µM) on MDA-MB231 cells.
