# Supplementary material for: Monitoring the Antioxidant Mediated Chemosensitization and ARE-Signaling in Triple Negative Breast Cancer Therapy
Source: PLoS One. 2015 Nov 4;10(11):e0141913. doi: 10.1371/journal.pone.0141913 (PMC4633093; doi:10.1371/journal.pone.0141913)
Supplement: S5 File — Apoptotic effect of antioxidant MMS (0–10 μM) in the presence or absence of anticancer drug cisplatin (15 μM) in MDA MB231 cells (Figure B in S1 File). (PDF) [file pone.0141913.s005.pdf]

**Supplementary Information Foygel et al.**

A

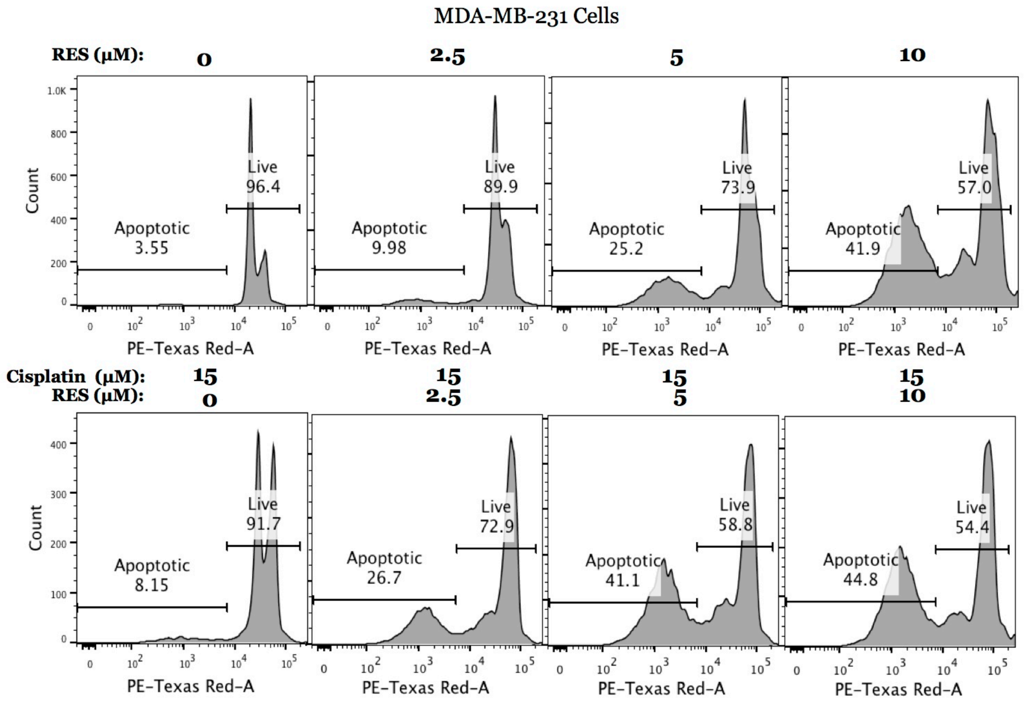


B

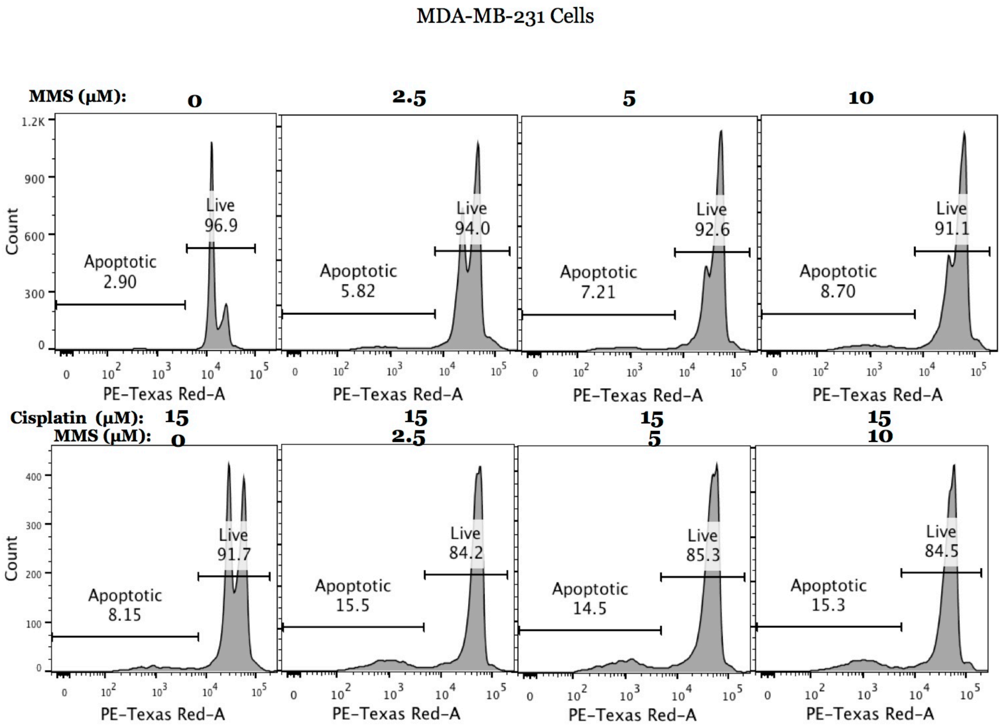


**S5 File. Apoptotic effect of antioxidant RES (0-10 μM) (Figure A) and MMS (0-10 μM) (FigureB) in the presence or absence of anticancer drug cisplatin (15 μM) in MDA MB231 cells.**
