# Supplementary material for: Monitoring the Antioxidant Mediated Chemosensitization and ARE-Signaling in Triple Negative Breast Cancer Therapy
Source: PLoS One. 2015 Nov 4;10(11):e0141913. doi: 10.1371/journal.pone.0141913 (PMC4633093; doi:10.1371/journal.pone.0141913)
Supplement: S6 File — Apoptotic effect of anticancer drug RRx-001 (2.5 μM) in response to antioxidant PTS (0–25 μM) on 4T1 cells (Figure B in S1 File). (PDF) [file pone.0141913.s006.pdf]

Supplementary Information Foygel et al.

A

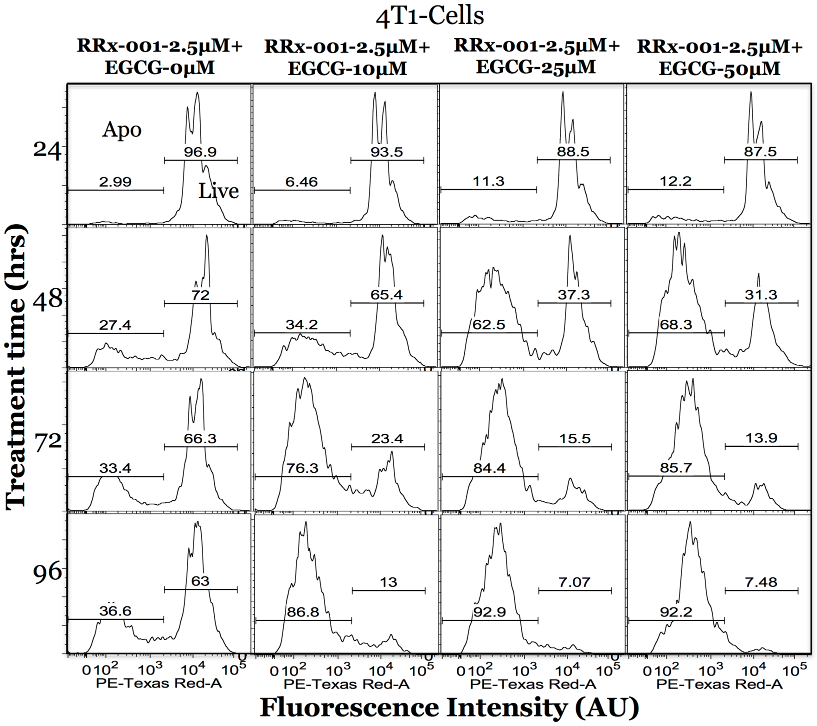

B

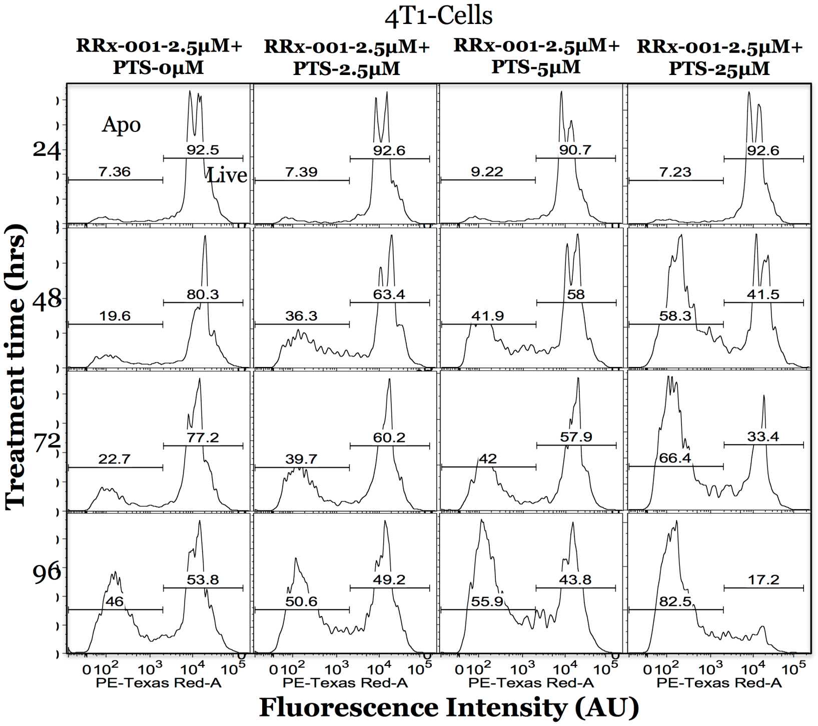

S6 File. Apoptotic effect of anticancer drug RRx-001 (2.5 μM) in response to antioxidant EGCG (0-50 μM) (Figure A) and PTS (0-25 μM) (Figure B) on 4T1 cells
