# Supplementary material for: Monitoring the Antioxidant Mediated Chemosensitization and ARE-Signaling in Triple Negative Breast Cancer Therapy
Source: PLoS One. 2015 Nov 4;10(11):e0141913. doi: 10.1371/journal.pone.0141913 (PMC4633093; doi:10.1371/journal.pone.0141913)
Supplement: S7 File — Effect of anticancer drug RRx-001 in response to antioxidant-Nrf2-activator EGCG in Mcf10a cells in combination with antioxidant EGCG (0–50 μM) (Figure B in S1 File). (PDF) [file pone.0141913.s007.pdf]

Supplementary Information Foygel et al.

A

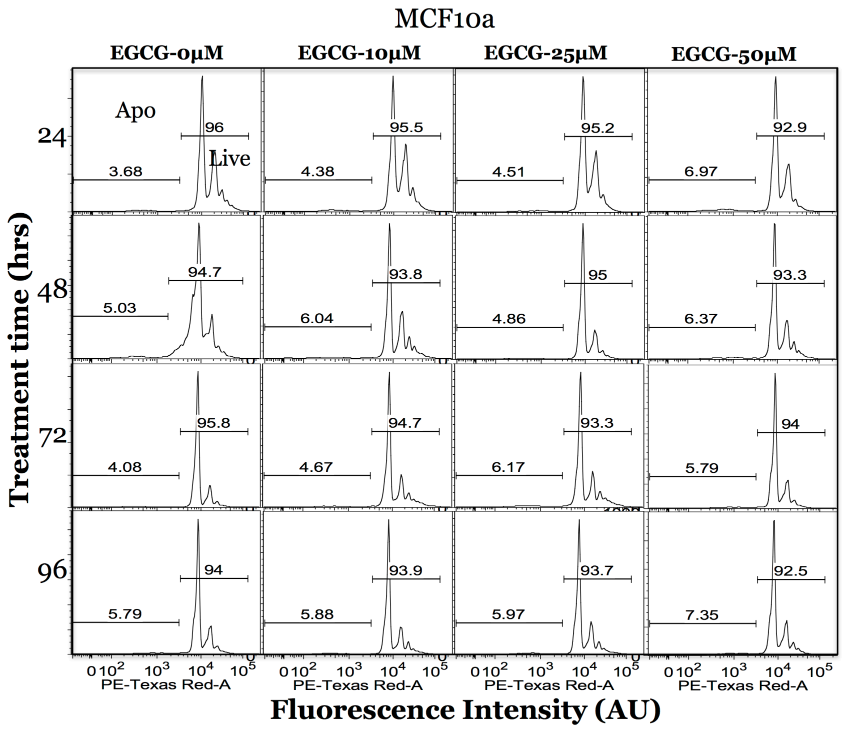

B

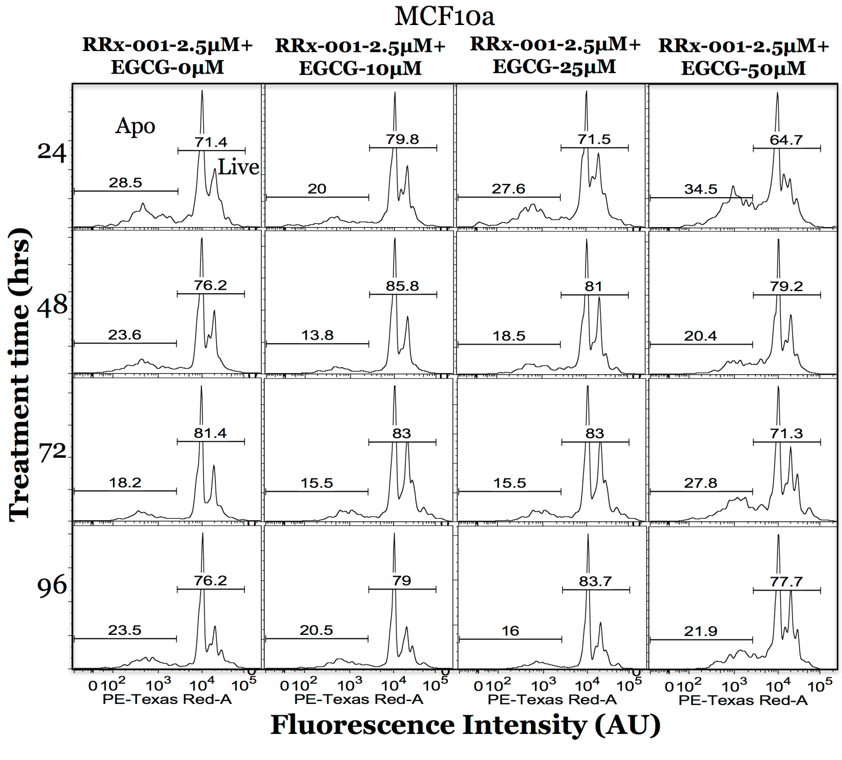

S7 File. Apoptotic effect of antioxidant EGCG (0-50 μM) in Mcf10a cells (Figure A). Effect of anticancer drug RRx-001 in response to antioxidant-Nrf2-activator EGCG in Mcf10a cells in combination with antioxidant EGCG (0-50 μM) (Figure B).
