# Supplementary material for: Monitoring the Antioxidant Mediated Chemosensitization and ARE-Signaling in Triple Negative Breast Cancer Therapy
Source: PLoS One. 2015 Nov 4;10(11):e0141913. doi: 10.1371/journal.pone.0141913 (PMC4633093; doi:10.1371/journal.pone.0141913)
Supplement: S8 File — Immunoblot analysis demonstrating Nrf2 expression in MDAMB231 cells treated with scrambled and Nrf2-specific siRNAs. Quantitative plot is shown on the right; error bars represent standard deviations of triplicate experiments (Figure B in S1 File). (PDF) [file pone.0141913.s008.pdf]

A

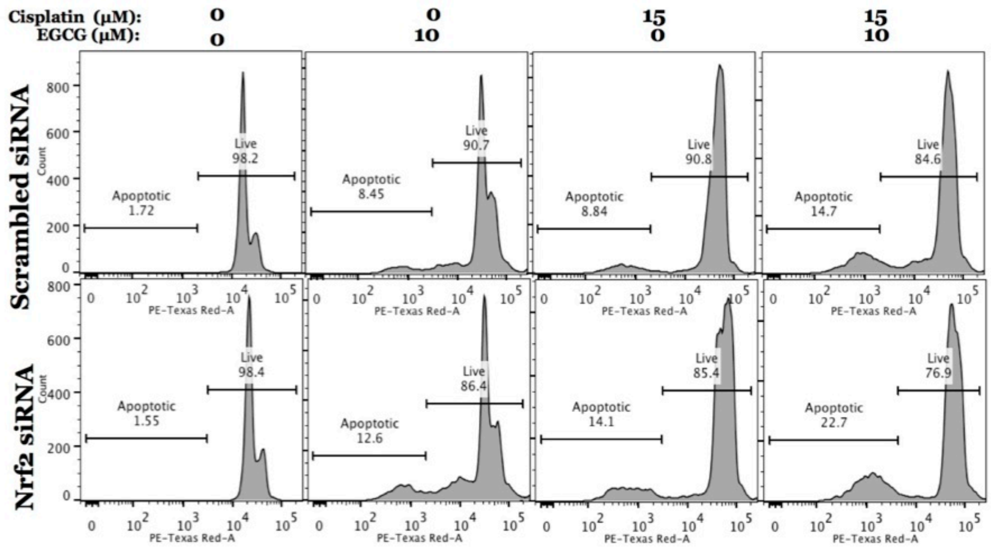

B

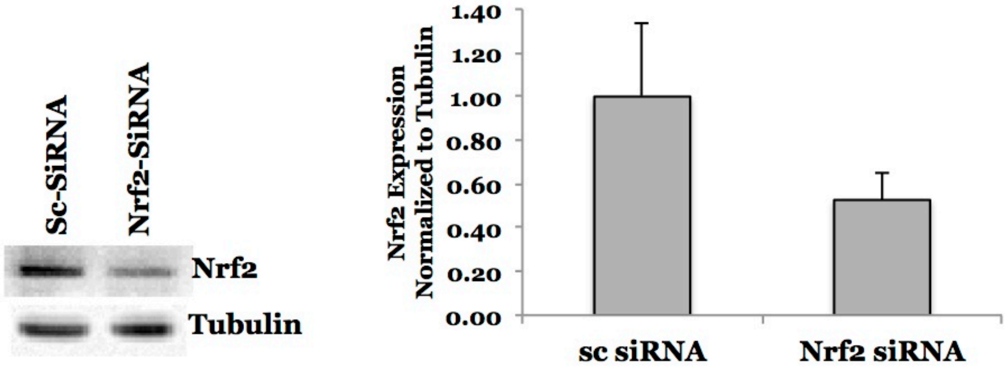

S8 Figure. Apoptotic effect of antioxidant EGCG (10 uM) in the presence and absence of chemotherapeutic drug cisplatin (15 μM) in MDA MB231 cells treated with Nrf2-specific or scrambled siRNA (Figure A). Immunoblot analysis demonstrating Nrf2 expression in MDAMB231 cells treated with scrambled and Nrf2-specific siRNAs. Quantitative plot is shown on the right; error bars represent standard deviations of triplicate experiments (Figure B).
